# Efficacy and Safety of Anticoagulants in Patients With Cirrhosis and Portal Vein Thrombosis: A Systematic Review and Meta‐Analysis of Randomized and Non‐Randomized Studies

**DOI:** 10.1002/jgh3.70194

**Published:** 2025-08-08

**Authors:** Muhammad Hassan Waseem, Zain ul Abideen, Marium Khan, Barka Sajid, Noor Ul Huda Ramzan, Rabeya Farid, Javed Iqbal, Jalib Ahmed, Aqsa Kabir, Maryam Shahzad, Javeria Asif, Muhammad Osama, Sania Aimen, Ammad Javaid Chaudhary, Ameer Haider Cheema

**Affiliations:** ^1^ Allama Iqbal Medical College Lahore Pakistan; ^2^ King Edward Medical University Neela Gumbad Lahore Pakistan; ^3^ Jinnah Sindh Medical University Karachi Pakistan; ^4^ University Medical and Dental College Faisalabad Pakistan; ^5^ Nursing Department Hamad Medical Corporation Doha Qatar; ^6^ Dow Medical College Karachi Pakistan; ^7^ Ziauddin Medical College Karachi Pakistan; ^8^ Khyber Medical College Peshawar Pakistan; ^9^ Quetta Institute of Medical Sciences Quetta Pakistan; ^10^ Henry Ford Hospital Detroit USA; ^11^ UT Southwestern Medical Center Dallas USA

**Keywords:** anticoagulants, liver cirrhosis, meta‐analysis, portal vein thrombosis, PVT

## Abstract

**Background:**

Portal vein thrombosis (PVT) contributes substantially to morbidity and mortality in cirrhotic patients. A clear insight into the anticoagulation therapy benefits in these patients could improve clinical decision‐making. This meta‐analysis aimed to assess the efficacy and safety of Anticoagulants in cirrhotic patients with PVT.

**Methods:**

PubMed, Cochrane Library, and ScienceDirect were searched from inception to September 2024. The Risk Ratios (RR) with 95% Confidence Interval (CI) were pooled for dichotomous outcomes under the random effects model using Review Manager 5.4.1. The primary endpoint of interest is PVT recanalization. Quality assessment was done through the Newcastle Ottawa Scale and the Cochrane RoB2.0 tool. Leave‐one‐out sensitivity analysis was done to investigate the cause of heterogeneity. Publication bias was assessed through funnel plots.

**Results:**

Twenty‐three studies (including 19 cohorts and 4 Randomized trials), pooling 81,599 patients, were included in the analysis. Anticoagulants significantly increased the PVT recanalization (RR = 2.00; 95% CI: [1.59, 2.52]; *p* < 0.00001; *I* ^2^ = 13%), PVT improvement (RR = 1.98; 95% CI: [1.70, 2.29], *p* < 0.00001; *I* ^2^ = 0%) while decreasing the PVT stability (RR = 0.78; 95% CI: [0.62,0.99], *p* = 0.04; *I* ^2^ = 19%) and PVT progression (RR = 0.42; 95% CI: [0.29, 0.60], *p* < 0.00001; *I* ^2^ = 27%). Other outcomes including mortality (RR = 0.53; 95% CI: [0.27, 1.03]; *p* = 0.06; *I* ^2^ = 94%), total bleeding (RR = 1.02; 95% CI: [0.76, 1.37], *p* = 0.89; *I* ^2^ = 31%), esophageal variceal bleeding (RR = 0.74; 95% CI: [0.54, 1.01], *p* = 0.06; *I* ^2^ = 56%), Gastrointestinal bleeding (RR = 1.07; 95% CI: [0.78, 1.48]; *p* = 0.66, *I* ^2^ = 13%) and Intracranial hemorrhage (RR = 1.19; 95% CI: [0.89, 1.58], *p* = 0.24, *I* ^2^ = 0%) were comparable between the 2 arms.

**Conclusion:**

Anticoagulants significantly increased PVT recanalization and PVT improvement while decreasing PVT stability and PVT progression in cirrhotic patients. Other outcomes were comparable between the two groups.

## 1 Introduction

Portal vein thrombosis (PVT) is a critical complication in patients with cirrhosis, characterized by the formation of a thrombus within the portal vein, leading to impaired hepatic blood flow. The prevalence of PVT has been rising, particularly among individuals with advanced liver disease, and is associated with poor outcomes, including increased risks of hepatic decompensation, variceal bleeding, and mortality [1].

Anticoagulation therapy has been proposed as a treatment strategy to promote the recanalization of the portal vein, prevent thrombus progression, and potentially improve clinical outcomes in cirrhotic patients. However, the application of anticoagulants in this population is contentious due to the elevated risk of bleeding complications inherent in patients with liver dysfunction. Despite these concerns, emerging evidence suggests that, in carefully selected patients, anticoagulation therapy may confer significant benefits, such as the prevention of clot progression, facilitation of partial or complete recanalization, and even the reduction of portal hypertension and improvement of liver function in some cases [2].

A variety of anticoagulants, including traditional agents like warfarin and low‐molecular‐weight heparin (LMWH), as well as newer direct oral anticoagulants (DOACs), have been used in the management of PVT in cirrhosis [3]. Nevertheless, the decision to initiate anticoagulation therapy is complex and must balance the risks of thrombotic events against the heightened susceptibility to bleeding complications, exacerbated by the underlying liver dysfunction.

Conflicting evidence regarding the efficacy and safety of anticoagulation in this context complicates clinical decision‐making. While some studies report benefits in achieving recanalization of the portal vein [4, 5], other research highlights significant concerns about increased risks of major bleeding, such as gastrointestinal (GI) hemorrhage and exacerbation of esophageal varices [6, 7]. The optimal duration and intensity of anticoagulation therapy remain subjects of debate, with factors such as the extent and chronicity of thrombosis, the presence of esophageal varices, and the severity of liver dysfunction influencing treatment decisions. Furthermore, the impact of anticoagulation on long‐term outcomes, including eligibility for liver transplantation and overall survival, requires further investigation through large‐scale, prospective clinical trials. While anticoagulation may offer potential benefits, it may not be appropriate for all cirrhotic patients with PVT, particularly those with advanced liver disease or existing esophageal varices, where the risks may outweigh the benefits.

Previous meta‐analyses on this topic have provided valuable insights but have been limited by heterogeneous patient populations, variations in study designs, and inconsistent outcome measures. By synthesizing data from recent studies, this systematic review and meta‐analysis seeks to clarify the impact of anticoagulation on critical clinical outcomes, thereby contributing to a more robust evidence base for informed clinical decision‐making.

## 2 Methodology

We conducted a systematic review and meta‐analysis following the Preferred Reporting Items for Systematic Review and Meta‐analyses (PRISMA) guidelines [8] and the Cochrane Handbook for Systematic Reviews of Interventions [9]. This review's protocol was pre‐registered on PROSPERO under the ID: CRD42024593233.

### 2.1 Search Strategy

An extensive literature search was performed across PubMed, Cochrane Library, and ScienceDirect databases starting from the beginning of each database up to August 2024. Keywords and Medical Subject Headings (MeSH) terms utilized in the search were “Liver Cirrhosis”, “Anticoagulants”, and “Portal vein thrombosis”. The relevant studies were identified by manually searching bibliographies. The detailed search strategy used for each electronic database is described in Table S1.

### 2.2 Study Screening and Eligibility Criteria

**FIGURE 1:**
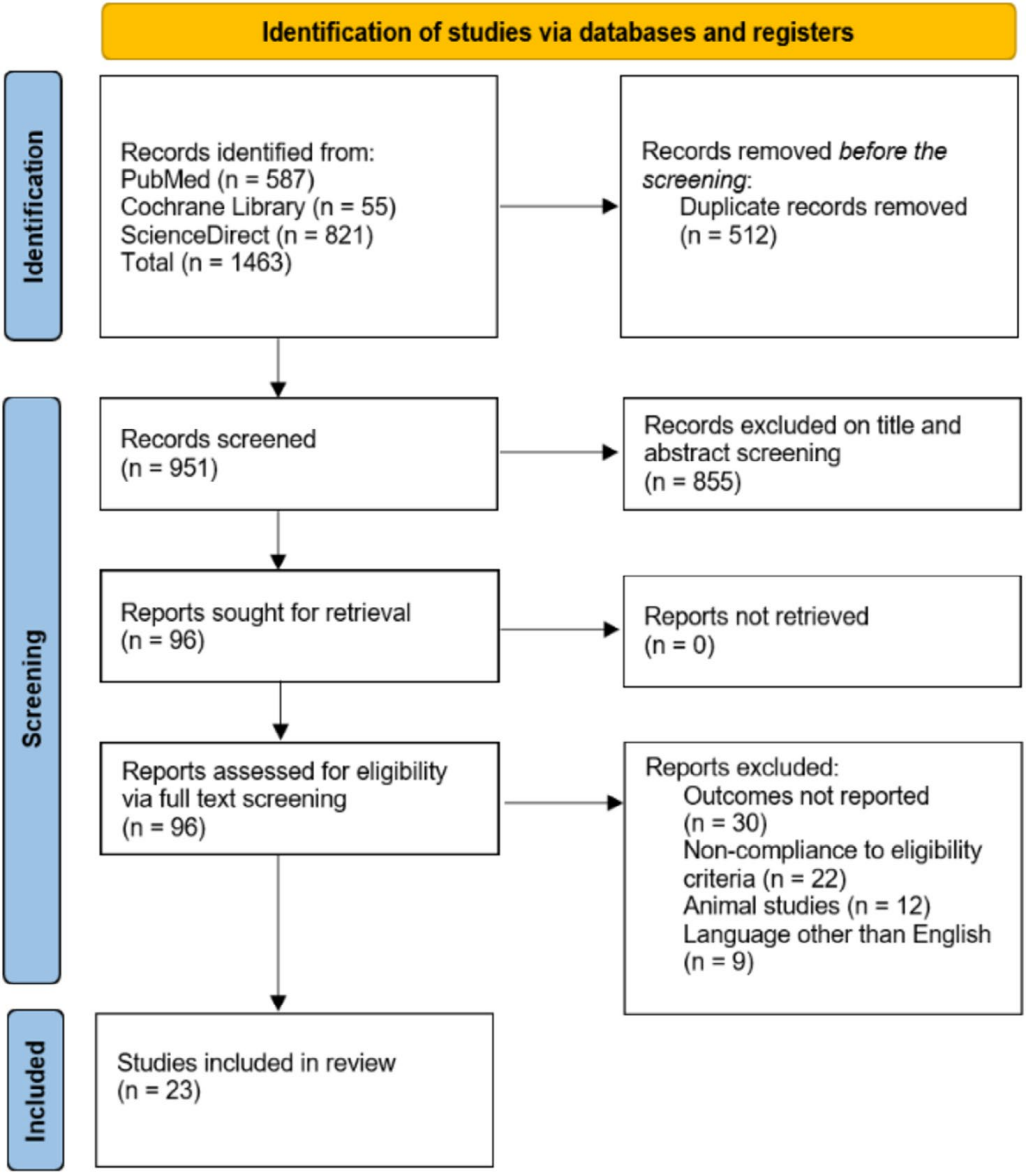
PRISMA flowchart of the study selection process.

Two reviewers (MK and MO) screened the titles and abstracts for related articles using Endnote version 20. A third reviewer (MHW) resolved the discrepancies. The complete texts of the remaining articles were reviewed to determine their eligibility based on the established criteria. Any conflicts or disagreements were resolved through the participation of a third author. Articles were only considered if they were retrospective or prospective full‐length research‐based studies that evaluated and compared the clinical efficacy of anticoagulation (including vitamin warfarin, LMWH, and DOACs) in managing PVT in cirrhosis. The criteria for patient selection and study exclusion are reported using PICOS (Patients, Intervention, Comparator, Outcomes, and Study Type). The inclusion criteria were studies that included adult (> 18 years) patients diagnosed with cirrhosis and PVT and examined the use of anticoagulation therapy (e.g., vitamin K antagonist (VKA) like warfarin, LMWH, DOACs, or a combination, antithrombin‐III (AT‐III) and nadroparin‐warfarin sequential therapy (NWS)) for the treatment of PVT. Those studies that had a non‐anticoagulative control group and reported the outcomes that were related to PVT resolution, PVT recanalization (partial or complete), all‐cause mortality, bleeding complications (including esophageal varices and GI bleeds), and other safety and efficacy outcomes were included. Randomized controlled trials (RCTs) and cohort studies were included. We included only those articles that were published in English. The exclusion criteria were studies involving pediatric patients or patients without a confirmed diagnosis of cirrhosis and those that did not specifically assess the impact of anticoagulation therapy on PVT. The studies that did not report on relevant efficacy or safety outcomes (e.g., PVT resolution, mortality, bleeding events), case reports, case series with fewer than 10 patients, review articles, editorials, and non‐comparative studies were excluded. We excluded the articles published in languages other than English. The detailed study selection process is depicted in the PRISMA flowchart Figure 1.

### 2.3 Data Extraction and Outcomes Definition

Data extraction was performed by two authors (JA and BS), with disparities being resolved by a third author (ZUA). A data extraction sheet was formulated, and relevant data were extracted and recorded from the included studies. The variables derived included: study characteristics (author, study duration, country, study design, publication year, and study population), patient characteristics (number of patients in each group, mean age, treatment group, time to follow up, and anticoagulation duration), and outcomes measured (number of patients in whom PVT recanalization was achieved (complete or partial), PVT improvement, PVT progression, mortality, number of patients who reported bleeding (Total, GI, or esophageal variceal) and intracranial hemorrhage). PVT was defined as thrombosis in the portal vein, including potential extension into intrahepatic branches and splenic or mesenteric veins, diagnosed by available imaging modalities. The complete resolution of fully occlusive PVT with transition to nonocclusive PVT is referred to as PVT recanalization. PVT improvement included both PVT recanalization and regression of PVT extension events. PVT progression included either further occlusion of the thrombus or extension of the thrombus to further segments of the superior mesenteric or splenic vein. All‐cause mortality was used, which meant death from any cause during the follow‐up period of the study.

### 2.4 Bias Assessment and Certainty of Evidence

Two reviewers (NUHR and AK) independently evaluated the quality of the included studies. The risk of bias in RCTs was evaluated using the Cochrane Risk‐of‐Bias Tool (RoB 2), [10] which assessed five domains as follows: randomization process, deviations from intended interventions, missing outcome data, outcome measurements, and selection of the reported results. The study was comprehensively screened and subsequently given a rating of low risk, high risk, or some concerns. Reasons for each rating were recorded. To assess the quality of the prospective and retrospective studies, the Newcastle‐Ottawa Scale (NOS) was utilized [11]. The primary domains of NOS are the selection of patients (score 0–4), comparability (score 0–2), and assessment of results (score 0–3). The maximum score is 9.

### 2.5 Statistical Analysis

The investigated outcomes were pooled together using Review Manager Software (RevMan, Version 5.4.1; The Cochrane Collaboration, Copenhagen, Denmark) [12]. Forest plots were generated to assess dichotomous data reported as risk ratio (RR) with 95% CI. A *p* value of ≤ 0.05 was deemed statistically significant for all the outcomes. Subgroup analysis was done based on the study design. The heterogeneity was assessed using the Higgins *I* ^2^ test [13], with substantial heterogeneity defined when *I* ^2^ is greater than 50% and requires further evaluation through sensitivity analyses using the leave‐one‐out technique.

## 3 Results

### 3.1 Search Results

1463 articles are retrieved by thoroughly searching databases like PubMed, ScienceDirect, and Cochrane Library. After removing duplicates (*n* = 512), we were left with 951 articles, which passed through the title and abstract screening process, yielding a total of 96 articles. These articles were then filtered through the full‐text screening, thus giving us a total of 23 studies [5, 14, 15, 16, 17, 18, 19, 20, 21, 22, 23, 24, 25, 26, 27, 28, 29, 30, 31, 32, 33, 34, 35] to be included in the final quantitative analysis.

### 3.2 Characteristics of Included Studies

This meta‐analysis pooled a total of 23 studies [5, 14, 15, 16, 17, 18, 19, 20, 21, 22, 23, 24, 25, 26, 27, 28, 29, 30, 31, 32, 33, 34, 35], including 19 observational studies [5, 14, 15, 16, 17, 18, 20, 21, 22, 23, 24, 26, 27, 29, 30, 31, 32, 34, 35] and four RCTs [19, 25, 28, 33]. These studies, published between 2012 and 2024, encompassed a total of 81 599 patients. The sample size ranged from 11 to 60 505, while the mean age ranged from 45 to 69 years. The follow‐up time and anticoagulation regimen duration ranged from 3 to 44 months and 1 to 19 months, respectively. Seven studies were conducted in China [15, 17, 24, 25, 28, 30, 33], 6 in the USA [5, 22, 27, 29, 34, 35], 2 in Japan [19, 32], and the rest 8 [14, 16, 18, 20, 21, 23, 26, 31] were from Italy, Romania, Portugal, Korea, Austria, and Singapore. The details of the baseline characteristics of the included studies are given in Table 1.

**TABLE 1:** Baseline characteristics of the included studies.

Authors	Location	Study design	Sample size (*n*)	Mean age years		Gender (M/F)	Intervention group	Anticoagulation Duration (months)	Follow‐up duration (months)
				Anticoagulation	Control				
Senzolo 2012	Italy and London	PC	56	55.5	52.3	38/18	LMWH	12	22.53
Cai 2013	China	RC	11	—	—	10/1	Warfarin, LMWH	3	37.6
Chung 2014	Korea	RC	28	59.4	58.7	21/7	Warfarin	3.7	3.8
Chen 2016	China	RC	66	44.97	47.86	47/19	Warfarin	7.6	33.2
Scheiner 2018	Austria	RC	51	—	—	32/19	LMWH, Phenprocoumon	12.0	44.1
Hidaka 2018	Japan	RCT	73	66.0	69.5	46/26	AT‐III	0.7	0.5
Sule 2018	Singapore	RC	30	55.6	66	19/11	Warfarin, LMWH	2.2	—
Acuna‐Villaorduna 2019	USA	RC	85	—	—	42/43	—	—	12
Ferreira 2019	Portugal	RC	80	59	60	53/27	Warfarin, LMWH	—	25.5
Mahmoudi 2019	USA	RC	60	—	—	42/9	Warfarin, LMWH (Dalteparin)	7.8	—
Pettinari 2019	Italy and Romania	RC	182	57.9	57.7	130/52	LMWH, Fondaparinux; VKA	13.4	19
Ai 2020	China	PC	80	56.1	52.3	50/30	DOACs (oral Rivaroxaban tablets or Dabigatran etexilate capsules)	6	3
Zhou 2020	China	RCT	64	55	53	42/22	Warfarin, LMWH (Nadroparin calcium)	5	6
Florescu 2021	Romania	RC	107	52.2	54.7	54/53	LMWH, VKA	—	32
Naymagon 2021	USA	RC	214	60.3	60	142/72	DOAC, Warfarin, LMWH (Enoxaparin)	18.8	27
Zhan 2021	USA	RC	38	62.4	60.7	28/10	Warfarin, LMWH (Enoxaparin), DOAC (Apixaban) and Fondaparinux	14.6	5.6
Gao 2023	China	RCT	86	56.54	55.58	50/36	LMWH (Nadroparin calcium), Warfarin	5	6
STarar 2022	USA	RC	60 505	59.8	59.9	38 721/21784	DOAC, Warfarin, LMWH (Nadroparin calcium)	—	—
Zhang 2023	China	RC	77	59.0	60.4	44/33	Warfarin, Rivaroxaban, Dabigatran, LMWH (Nadroparin) Edoxaban	6	26
MANTAKA 2023	Greece	RC	76	—	—	—	VKA, LMWH	—	6
Sato 2023	Japan	RC	42	67.6	69.7	31/11	DOAC, Warfarin, Heparin	—	—
Lu 2024	China	RCT	64	53.8	53.4	22/42	Warfarin, LMWH (nadroparin calcium)	—	12
Niu 2024	USA	RC	19 524	58.3	57.0	10 775/8224	VKA and DOAC	—	—

Abbreviations: AT‐III, antithrombin III; DOAC, direct oral anticoagulant; LMWH, low molecular weight heparin; M/F, male/female; PC, prospective cohort; RC, retrospective cohort; RCT, randomized controlled trial; VKA, vitamin k antagonist.

### 3.3 Risk of Bias and Quality Assessment

The quality of the observational cohort studies were assessed through the NOS [11]. Seven observational studies were of high quality with NOS scores of 8 and 9. The remaining 12 studies were of moderate quality, with NOS scores ranging from 6 to 7. For RCTs, the Cochrane RoB 2.0 tool [10] was used to assess the quality. All the RCTs have a low risk of bias. The inclusion of both high and moderate quality observational cohort studies, along with the high quality RCTs, enhances the overall reliability of our findings. Although moderate‐quality studies introduce some degree of uncertainty, their presence in the pooled analysis contributes to a broader understanding of the evidence, with high‐quality observational studies and RCTs providing significant support, enhancing the robustness of our results. Details of the quality assessment of the cohorts and RCTs were given in Table S2 and Figure S1.

## 4 Outcomes

### 4.1 PVR Recanalization

**FIGURE 2:**
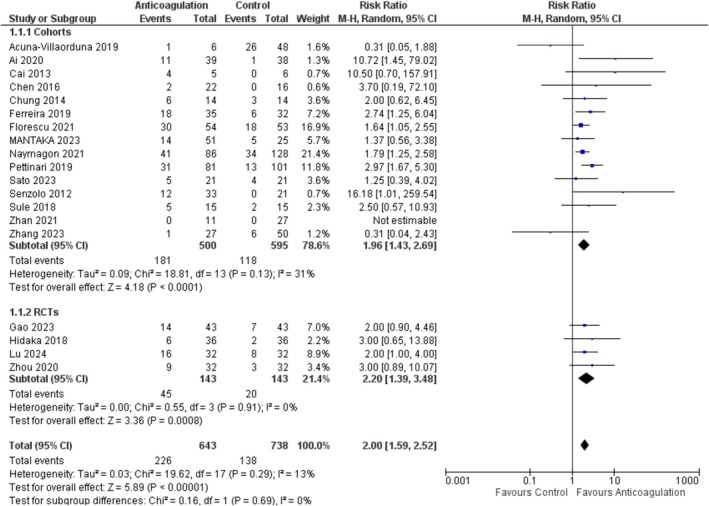
Portal vein thrombosis recanalization forest plot.

A pooled analysis of 19 studies [5, 14, 15, 16, 17, 19, 20, 21, 23, 24, 25, 26, 27, 28, 30, 31, 32, 33, 35], with a total of 1381 patients (643 anticoagulation and 738 placebo), revealed a statistically significant increase in the rate of PVT recanalization as compared to the control (RR = 2.00; 95% CI: [1.59, 2.52]; *p* < 0.00001; *I* ^2^ = 13%). On subgroup analysis based on study designs, both subgroups showed the same trend of increase in PVT recanalization Figure 2. On subgroup analysis based on the type of anticoagulant, the results became non‐significant for the AT‐III group (RR = 3.00; 95% CI: [0.65, 13.88]; *p* = 0.16) while for other subgroups the increase in recanalization remained significant Figure S2.

### 4.2 PVT Improvement

**FIGURE 3:**
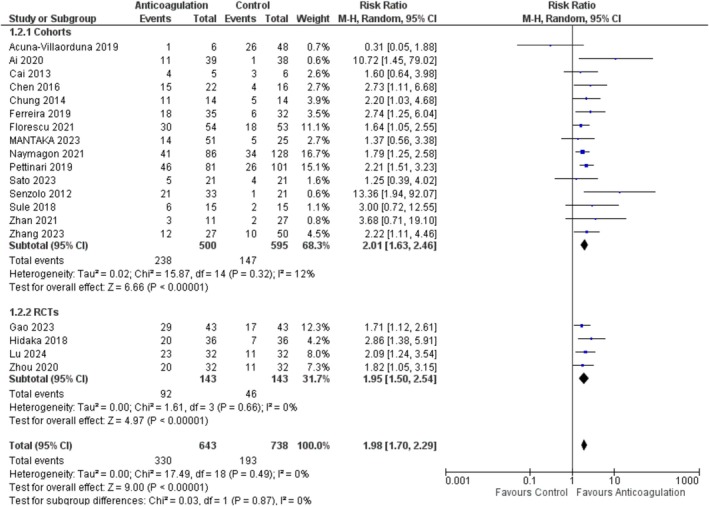
Portal vein thrombosis improvement forest plot.

The outcome, Portal venous thrombosis improvement, was reported by 19 studies [5, 14, 15, 16, 17, 19, 20, 21, 23, 24, 25, 26, 27, 28, 30, 31, 32, 33, 35], comprising 1381 patients (643 anticoagulation, and 738 placebo). The overall pooled analysis revealed that anti‐coagulation improved portal vein thrombosis, and this finding was statistically significant (RR = 1.98; 95% CI: [1.70, 2.29], *p* < 0.00001; *I* ^2^ = 0%). Subgroup analysis by study design did not reveal significant differences, with each group individually agreeing with the pooled findings Figure 3. Similarly, on subgroup analysis based on anticoagulant type, the results remained significant for each subgroup Figure S3.

### 4.3 PVT Stability

**FIGURE 4:**
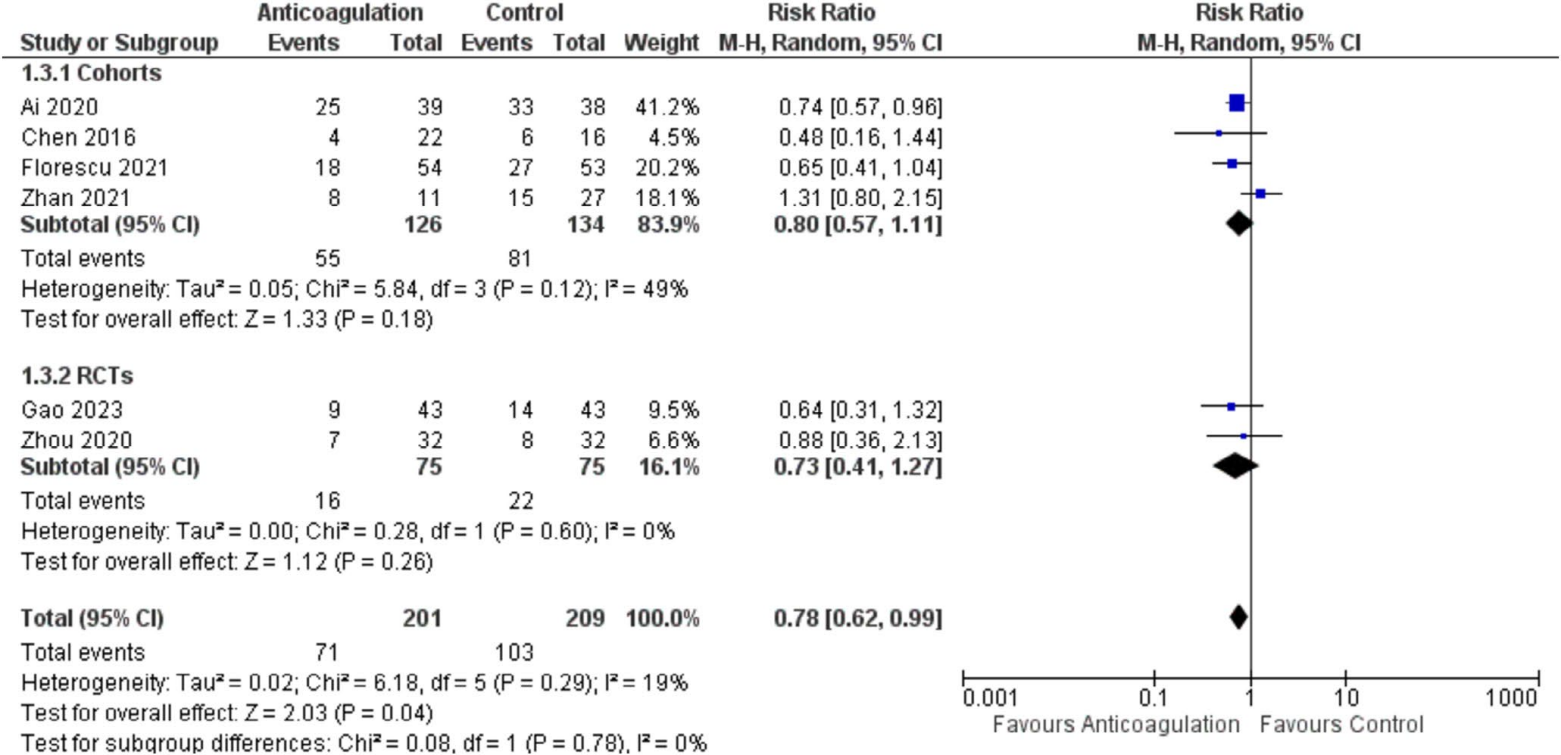
Portal vein thrombosis stability forest plot.

A pooled analysis of six studies [17, 24, 25, 26, 28, 35], with 410 patients (201 anticoagulation, and 209 placebo), revealed statistically significant results in favor of anticoagulation as compared to control (RR = 0.78; 95% CI: [0.62, 0.99], *p* = 0.04; *I* ^2^ = 19%). On subgroup analysis by study design, the results became non‐significant for both the subgroups Figure 4. On subgroup analysis by anticoagulant type, the results became non‐significant for all the subgroups with the exception of DOACs, which caused a significant decrease in the PVT stability (RR = 0.74; 95% CI: [0.57, 0.96], *p* = 0.02) Figure S4.

### 4.4 PVT Progression

**FIGURE 5:**
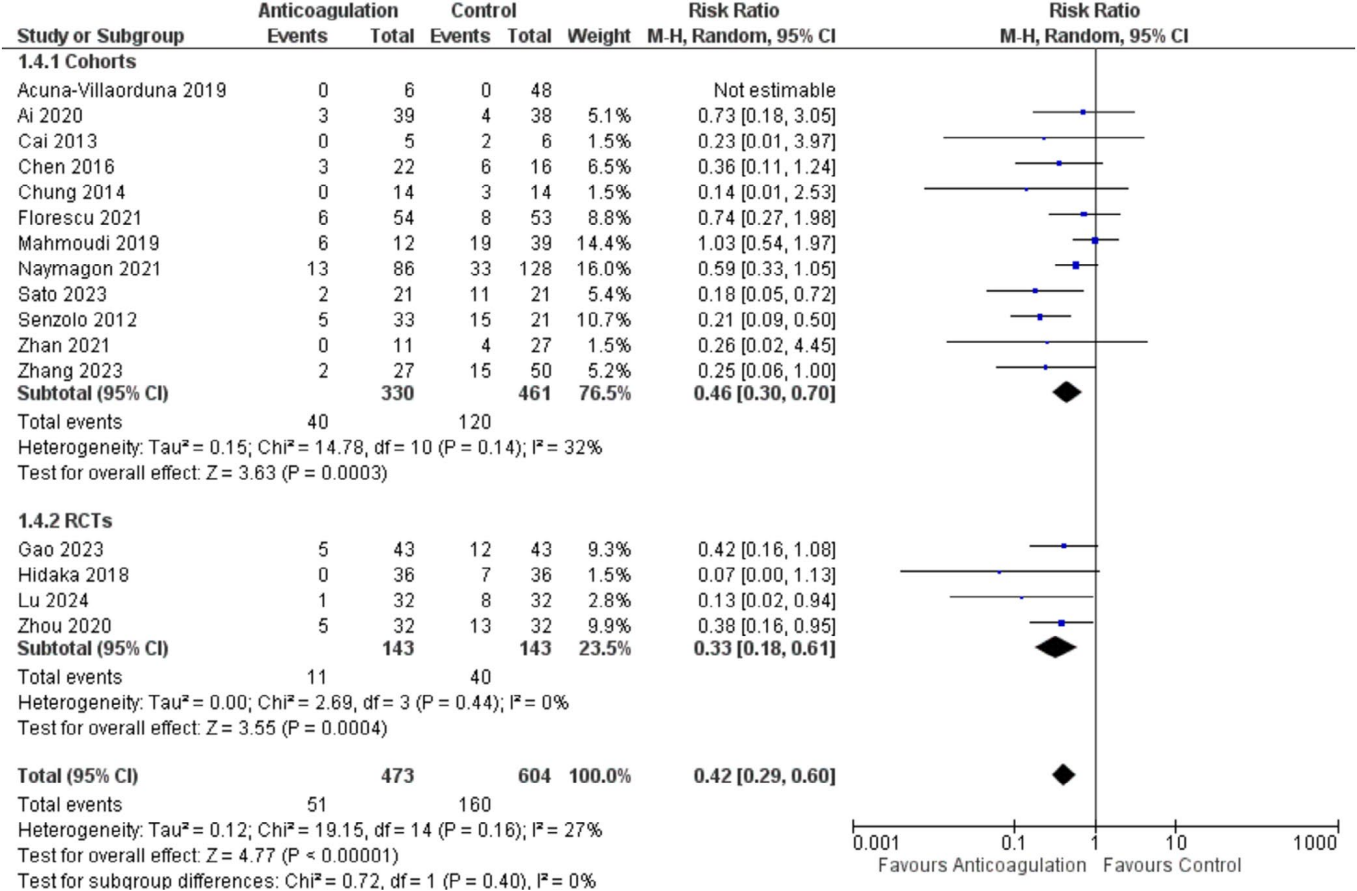
Portal vein thrombosis progression forest plot.

A total of 16 studies [5, 14, 15, 16, 17, 19, 22, 24, 25, 26, 27, 28, 30, 32, 33, 35] reported the progression of PVT among 1077 patients (473 anticoagulation, and 604 placebo). The pooled analysis of these studies revealed a statistically significant decreased risk of PVT progression with anticoagulation as compared to control (RR = 0.42; 95% CI: [0.29, 0.60], *p* < 0.00001; *I* ^2^ = 27%). Sub‐group analysis based on the study design also showed a decreased risk of progression with anticoagulation in both subgroups Figure 5. Whereas on subgrouping based on anticoagulant type, all the subgroups showed a significant decrease in PVT progression except for the AT‐III (RR = 0.07; 95% CI: [0.00, 1.13], *p* = 0.06) and DOACs (RR = 0.73; 95% CI: [0.18, 3.05], *p* = 0.67) groups, Figure S5.

### 4.5 Mortality

**FIGURE 6:**
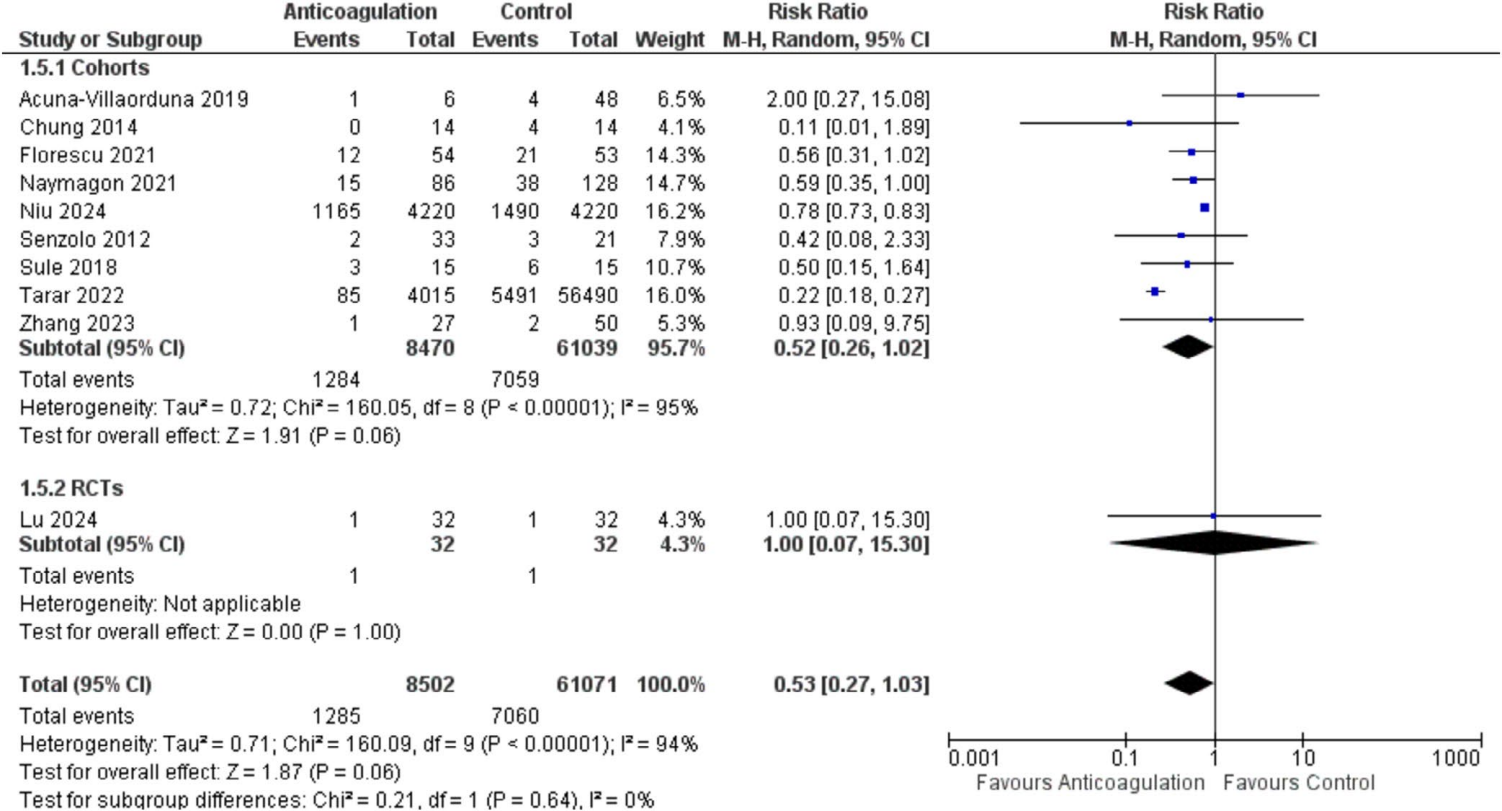
Mortality forest plot.

A pooled analysis of 10 studies [5, 14, 16, 20, 26, 27, 29, 30, 33, 34], with a total of 69 573 patients (8502 anticoagulation, and 61 071 placebo), revealed no statistically significant difference in mortality between the two groups (RR = 0.53; 95% CI: [0.27, 1.03]; *p* = 0.06). There was significant heterogeneity between the studies (*I* ^2^ = 94%). On subgroup analysis based on the study design, the results remained non‐significant for both subgroups Figure 6. Similarly, subgrouping based on the anticoagulant type also revealed a non‐significant trend Figure S6.

### 4.6 Total Bleeding

The event of bleeding was reported by 15 studies [14, 15, 16, 20, 23, 24, 25, 26, 27, 28, 29, 30, 32, 33, 35], comprising 61 578 patients(4508 anticoagulation, and 57 070 placebo). Pooled analysis of these studies revealed no change in risk of bleeding with anticoagulation as compared to control; statistically, this result was nonsignificant (RR = 1.02; 95% CI: [0.76, 1.37], *p* = 0.89, *I* ^2^ = 31%). Subgroup analysis by study design also revealed comparable results between the two groups Figure S7. Similarly, subgrouping based on the anticoagulant type also showed nonsignificant results Figure S8.

### 4.7 Esophageal Variceal Bleeding

A pooled analysis of 13 studies [14, 15, 16, 23, 24, 25, 26, 27, 28, 29, 30, 32, 34], with a total of 69 886 patients (8670 anticoagulation, and 61 216 placebo) revealed that the risk of esophageal variceal bleeding in a PVT patient was comparable between the anticoagulants and placebo (RR = 0.74; 95% CI: [0.54, 1.01], *p* = 0.06). There was moderate heterogeneity between the studies, *I* ^2^ = 56%. On subgroup analysis based on the study design, the results remained non‐significant for both subgroups Figure S9. Similarly, on subgrouping based on the anticoagulant type, the results remained comparable between the anticoagulants and placebo for all the subgroups Figure S10.

### 4.8 GI Bleeding

Six studies [23, 24, 25, 32, 33, 34] reported event rates for GI bleeding in patients with portal vein thrombosis, and pooled analysis of 8869 patients (4425 anticoagulation, and 4444 placebo) revealed no significant risk reduction with anticoagulation (RR = 1.07; 95% CI: [0.78, 1.48]; *p* = 0.66, *I* ^2^ = 13%). On subgroup analysis based on the study design, the results remained non‐significant Figure S11. Similarly, subgrouping based on the anticoagulant type also showed comparable risk of GI bleeding in all the subgroups Figure S12.

### 4.9 Intracranial Hemorrhage

Pooled analysis from four studies [16, 32, 33, 34] comprising 8574 patients (4287 anticoagulation, and 4287 placebo) showed a comparable risk of intracranial hemorrhage between the anticoagulants and placebo (RR = 1.19; 95% CI: [0.89, 1.58], *p* = 0.24, *I* ^2^ = 0%). On subgrouping by study design, the results remained non‐significant for both the subgroups Figure S13. Similarly, on subgroup analysis based on the anticoagulant type, the risk of intracranial hemorrhage between the anticoagulants and placebo remained comparable in all the subgroups Figure S14.

### 4.10 Sensitivity Analysis

A leave‐one‐out sensitivity analysis was conducted to evaluate how the study sample size affects the overall results, aiming to identify if smaller studies or those with larger sample sizes had a disproportionate influence on the findings. On removing the study by Niu 2024 [34] the heterogeneity decreased from 56% to 17% in esophageal variceal bleeding Figure S15. Similarly, on removing the study by Tarar 2022 [29] the heterogeneity decreased from 94% to 0% in the mortality outcome Figure S16. Overall, the findings of the meta‐analysis were robust, and no single study significantly impacted the study results.

### 4.11 Publication Bias

Publication bias was assessed visually through a funnel plot and statistically through Egger's regression. No publication bias was detected except for PVT progression, which shows statistically significant (*p* = 0.01404) bias on Egger's regression. The funnel plots of primary and secondary outcomes are depicted in Figure S17–S25.

## 5 Discussion

The prevalence of PVT associated with liver cirrhosis is about 13.9%, which continues to amplify with the severity of the disease and contributes to a yearly factor of about 10.4% [36]. Administration of anti‐coagulants for acute PVT has remained a standard treatment of choice, but their effectiveness in treating cirrhotic patients suffering from PVT remains unclear, with limited evidence‐based guidelines for its standard care [37]. Currently approved anti‐coagulants in cirrhosis include Vitamin K antagonists (VKA) such as Warfarin, LWMH, and DOACS such as Rivaroxaban, Dabigatran, Edoxaban, etc. [38]. The rationale of this analysis was driven by the fact that previously conducted studies have focused on the comparative analysis of several anti‐coagulants in addition to no anti‐coagulant therapies, but none have performed a complete assessment of anti‐coagulant therapies relative to a placebo drug. By incorporating the studies that have compared these interventions with no anticoagulative therapy or placebo, this meta‐analysis has provided an augmented evaluation of their clinical significance in cases where these therapies are not administered. This approach is necessary to assist clinicians in making well‐informed choices based on a thorough evaluation of the associated benefits of these interventions.

The novelty of our study lies in the fact that it included a broader range of anticoagulants (LMWH, warfarin, DOACs), enhancing the generalizability of the findings. The larger sample size and the inclusion of recent studies enhance the statistical power and strength of these findings and provide up‐to‐date evidence for clinical decision‐making.

The PVT recanalization being the primary endpoint showed a higher inclination towards the anti‐coagulant group, which not only aligns with the results of the previously conducted meta‐analysis but also with the American Gastroenterological Association (AGA) Clinical Practice guidelines that highlighted the treatment with Warfarin or Heparin derived medications was linked to higher rates of recanalization [39, 40, 41]. It also revealed notable improvements in the partial and complete recanalization of PVT. This association could be drawn due to the anti‐coagulant's ability to prevent clotting factors thus preventing the probability of further thrombus formation as well as aiding in the reduction of the clot size which can then lead to an improved portal vein blood flow. This is also reiterated by the results of a retrospective cohort, according to which anti‐coagulants enhanced the rate of PVT recanalization without affecting the risk of bleeding in addition to potentially declining the rate of variceal bleeding [30]. Another cohort study reported that 75.9% of their patient population who received anti‐coagulant therapy were successful in thrombus recanalization, additionally confirming anticoagulation as a key determinant of PVT recanalization [42]. According to the European Association for the Study of the Liver's (EASL) clinical practice guideline, cirrhotic patients suffering from PVT should be considered for anticoagulation at therapeutic doses for at least 6 months [43].

Our analysis revealed a significant decline in the progression/extension and stability of PVT when administered with anti‐coagulants in comparison to the patients who did not receive any anti‐coagulants. Anti‐coagulants' ability to promote thrombus resolution and its formation can greatly contribute to its reduced stability and progression. Limiting the thrombus's expansion towards other areas by preventing its further growth can spare a great deal of PVT progression, which is highlighted by our results. Halting PVT's extension prevents various major complications such as portal hypertension, intestinal infarction, variceal bleeding, hepatic encephalopathy, and so on [44]. The findings of our study align with the previous meta‐analysis such as Qi et al. 2015, and Yao et al. 2023 which also state decreased PVT progression in patients treated with anti‐coagulants [40, 45]. Fibrin plays an important role in clots by binding to activated platelets through specific receptors such as integrin αIIbβ3, which in turn strengthens platelets to adhere, hence stabilizing the clot [46]. Moreover, fibrin also interacts with a platelet collagen receptor known as GPVI, which in turn enhances the exposure of Phosphatidylserine, which is an important component in clot growth and stability [46]. Anti‐coagulants disrupt these processes by inhibiting platelet aggregation as well as fibrin binding, which in turn, negatively affects the clot's stability, leading to its breakdown.

The forest plot comparing the incidence of mortality in either group demonstrates rather statistically insignificant results, which, to some extent, could signify no evidence‐based benefit or harm caused by anticoagulants. Although studies by Guerrero et al. (2023) and Zaman et al. (2019) reported that anticoagulant therapy improved the prognosis of patients with PVT, our meta‐analysis showed no statistically significant difference in mortality between the anticoagulant and non‐anticoagulant groups (RR = 0.53; 95% CI: [0.27, 1.03], *p* = 0.06) [36, 40, 47]. However, our findings still suggest a decreasing trend in mortality in the anticoagulant group. This discrepancy may be due to variations in study designs, patient characteristics, anticoagulant types, and follow‐up durations. The results highlight the need for more large‐scale, long‐term studies with consistent treatment protocols to confirm the potential benefits of anticoagulation in improving survival for cirrhotic patients with PVT. With early diagnosis and treatment, there is a chance of an 85% five‐year survival rate with anticoagulant therapy, with an enhanced prognosis in an acute condition than a chronic PVT, where the liver is majorly affected [48].

An increase in the bleeding events poses a major risk during the administration of anticoagulants in cirrhotic patients with PVT. The results of our meta‐analysis revealed no statistically significant value for the outcomes of total, esophageal variceal, and GI bleeding in the group treated with anti‐coagulant therapy when compared to the group managed with no anti‐coagulants [48]. This evidence is consistent with the previously conducted meta‐analyses, which revealed no increased risk of bleeding with the use of anti‐coagulants [39, 40]. While the studies by Loffredo et al. (2017) and Gao et al. (2021) found lower rates of esophageal variceal bleeding in anticoagulant‐treated patients, our meta‐analysis, which includes a larger and more diverse group of studies, showed a non‐significant trend towards reduced bleeding (RR = 0.74; 95% CI [0.54, 1.01], *p* = 0.06) [49, 50]. This discrepancy may be due to variations in patient populations and anticoagulant types. Our findings suggest that while anticoagulants may offer benefits, further studies with more consistent protocols and patient groups are needed to confirm these effects. Moreover, due to the utilization of various types of anti‐coagulants, that is, LMWH, VKA, DOAC, across the included studies, we were unable to assess the effects of individual anticoagulants on the bleeding outcomes. Regardless, our study supports the management of cirrhotic patients with PVT undergoing anti‐coagulant therapy with careful monitoring and assessment of each individual based on their current conditions, with reversal agents on board to cater to the bleeding complications [51]. Moreover, the American College of Gastroenterology (ACG) guidelines state that gastroesophageal varices are not a contraindication to anticoagulant therapy use, as it does not elevate the risk of variceal bleeding in cirrhotic patients with PVT [52].

In addition, intracranial hemorrhage in both groups aggregates a statistically insignificant difference in our study. Few of the previous meta‐analyses do not report this complication as one of the outcomes [39, 40, 45]. The overall effect could have been influenced due to the varying pharmacokinetic properties of each anti‐coagulant as evidenced by a meta‐analysis by Ruff et al. 2014 [53].

This comprehensive systematic review and meta‐analysis has certain strengths, like drawing a comparison between the use of anticoagulants to patients receiving no anticoagulation across various populations from multiple countries with different clinical settings as well as duration. This could potentially assist with increasing the generalizability of the findings to a wider patient group. The study incorporates various types of anticoagulants, which enhances the robustness of the investigated outcomes to utilize in the treatment of cirrhotic patients suffering from PVT. Moreover, it allows gathering evidence of comparative understanding of their effectiveness and safety profile.

The study also comes with its limitations. The inclusion of a substantial number of observational studies in comparison to RCTs can potentially reduce the overall strength of the evidence. Different anticoagulants such as DOACs, VKA‐like warfarin, LMWH, AT‐III, and NWS have different modes of action, efficacy, and safety profiles, so grouping them might obscure their safety and risk profile, but we have done subgroup analysis based on the anticoagulant types to address this limitation. Furthermore, the interpretation of long‐term outcomes may be impacted by the variability in follow‐up periods among the included studies. To increase the sample size and capture a wider range of patient experiences, studies with different follow‐up periods had to be included; nevertheless, it is crucial to recognize that this diversity may restrict the generalizability of findings regarding long‐term outcomes. Future studies with more consistent follow‐up durations will help clarify the long‐term benefits and risks of anticoagulation therapy in cirrhotic patients with portal vein thrombosis. Many of the studies included in our meta‐analysis did not provide detailed information on key aspects such as dose intensities, timing of dose modifications, or specific causes of treatment discontinuation, which limited our ability to include these details in our analysis. Additionally, while some studies used hepatic arterial infusion chemotherapy (HAIC), technical information like the site of the indwelling catheter and management protocols was often insufficiently documented. This highlights a significant gap in the literature, and we recommend that future studies address these aspects to improve clinical decision‐making and treatment outcomes.

## 6 Conclusion

As per the findings of our study, the anti‐coagulants are effective in PVT recanalization as well as resolution rates, but there are clinically insignificant results in terms of bleeding assessment in the patient population. Therefore, there is a need to conduct more clinical trials in the future, including newer anticoagulants, incorporating a variable patient population to collect robust results for treatment guidelines and options for cirrhotic patients with PVT.

## Ethics Statement

The authors have nothing to report.

## Consent

The authors have nothing to report.

## Conflicts of Interest

The authors declare no conflicts of interest.

## Supporting information


**Table S1.** Detailed Search Strategies Used in Different Databases.
**Table S2.** Newcastle Ottawa Scale for the quality assessment of the non‐randomized studies.
**Figure S1.** Quality assessment of the RCTs by RoB 2.0 tool.
**Figure S2.** Subgroup analysis based on anticoagulant type for PVT recanalization.
**Figure S3.** Subgroup analysis based on anticoagulant type for PVT improvement.
**Figure S4.** Subgroup analysis based on anticoagulant type for PVT stability.
**Figure S5.** Subgroup analysis based on anticoagulant type for PVT progression.
**Figure S6.** Subgroup analysis based on anticoagulant type for mortality.
**Figure S7.** Total Bleeding Forest plot.
**Figure S8.** Subgroup analysis based on anticoagulant type for total bleeding.
**Figure S9.** Esophageal variceal bleeding Forest plot.
**Figure S10.** Subgroup analysis based on anticoagulant type for Esophageal variceal bleeding.
**Figure S11.** Gastrointestinal Bleeding Forest plot.
**Figure S12.** Subgroup analysis based on anticoagulant type for GI bleeding.
**Figure S13.** Intracranial Hemorrhage Forest plot.
**Figure S14.** Subgroup analysis based on anticoagulant type for intracranial hemorrhage.
**Figure S15.** Esophageal variceal bleeding leave‐one‐out sensitivity analysis plot.
**Figure S16.** Mortality leave‐one‐out sensitivity analysis plot.
**Figure S17.** Portal Vein Thrombosis Recanalization Funnel plot.
**Figure S18.** Portal Vein Thrombosis Improvement Funnel plot.
**Figure S19.** Portal Vein Thrombosis Stability Funnel plot.
**Figure S20.** Portal vein thrombosis progression Funnel plot.
**Figure S21.** Mortality Funnel plot.
**Figure S22.** Total Bleeding Funnel plot.
**Figure S23.** Esophageal variceal bleeding Funnel plot.
**Figure S24.** Gastrointestinal Bleeding Funnel plot.
**Figure S25.** Intracranial Hemorrhage Funnel plot.

## References

[1] S. Prakash , J. Bies , M. Hassan , A. Mares , and S. C. Didia , “Portal Vein Thrombosis in Cirrhosis: A Literature Review,” Front Med (Lausanne) 10 (2023): 10. 10.3389/fmed.2023.1134801 PMC10169608 37181351

[2] L. Wang , X. Guo , X. Xu , et al., “Anticoagulation Favors Thrombus Recanalization and Survival in Patients With Liver Cirrhosis and Portal Vein Thrombosis: Results of a Meta‐Analysis,” Advances in Therapy 38 (2021): 495–520. 33155180 10.1007/s12325-020-01550-4 PMC7854392

[3] S. Gupta , J. Hidalgo , B. Singh , et al., “Usage of Direct Acting Oral Anticoagulants in Cirrhotic and Non‐Cirrhotic Portal Vein Thrombosis: A Systematic Review,” Cureus 13, no. 8 (2021): e16922. 34367844 10.7759/cureus.16922 PMC8342267

[4] M. Senzolo , N. Riva , F. Dentali , et al., “Long‐Term Outcome of Splanchnic Vein Thrombosis in Cirrhosis,” Clinical and Translational Gastroenterology 9, no. 8 (2018): 176. 30108204 10.1038/s41424-018-0043-2 PMC6092393

[5] A. Acuna‐Villaorduna , V. Tran , J. D. Gonzalez‐Lugo , E. Azimi‐Nekoo , and H. H. Billett , “Natural History and Clinical Outcomes in Patients With Portal Vein Thrombosis by Etiology: A Retrospective Cohort Study,” Thrombosis Research 174 (2019): 137–140. 30597344 10.1016/j.thromres.2018.12.019

[6] F. Nery , S. Chevret , B. Condat , et al., “Causes and Consequences of Portal Vein Thrombosis in 1,243 Patients With Cirrhosis: Results of a Longitudinal Study,” Hepatology 61 (2015): 660–667. 25284616 10.1002/hep.27546

[7] K. Berry , J. Taylor , I. W. Liou , and G. N. Ioannou , “Portal Vein Thrombosis Is Not Associated With Increased Mortality Among Patients With Cirrhosis,” Clinical Gastroenterology and Hepatology 13 (2015): 585–593. 25459555 10.1016/j.cgh.2014.10.010

[8] M. J. Page , J. E. McKenzie , P. M. Bossuyt , et al., “The PRISMA 2020 Statement: An Updated Guideline for Reporting Systematic Reviews,” BMJ (Clinical Research Ed.) 372 (2021): n71. 10.1136/bmj.n71 PMC8005924 33782057

[9] J. P. T. Higgins , J. Thomas , J. Chandler , et al., Cochrane Handbook for Systematic Reviews of Interventions (John Wiley & Sons, 2019), 1–694.

[10] J. A. C. Sterne , J. Savović , M. J. Page , et al., “RoB 2: A Revised Tool for Assessing Risk of Bias in Randomised Trials,” BMJ (Clinical Research Ed.) 366 (2019): l4898. 10.1136/bmj.l4898 31462531

[11] G. FC , R. Marcos , and A. RAN , “The Newcastle‐Ottawa Scale (NOS) for Assessing the Quality of Nonrandomized Studies,” PLoS Neglected Tropical Diseases (2013).

[12] RevMan , “Cochrane Training Trusted Evidence. Informed Decisions,” Better Health.

[13] J. P. T. Higgins , S. G. Thompson , J. J. Deeks , and D. G. Altman , “Measuring Inconsistency in Meta‐Analyses,” BMJ 327 (2003): 557–560. 12958120 10.1136/bmj.327.7414.557 PMC192859

[14] M. Senzolo , T. M. Sartori , V. Rossetto , et al., “Prospective Evaluation of Anticoagulation and Transjugular Intrahepatic Portosistemic Shunt for the Management of Portal Vein Thrombosis in Cirrhosis,” Liver International 32 (2012): 919–927. 22435854 10.1111/j.1478-3231.2012.02785.x

[15] M. Cai , K. Zhu , W. Huang , et al., “Portal Vein Thrombosis After Partial Splenic Embolization in Liver Cirrhosis: Efficacy of Anticoagulation and Long‐Term Follow‐Up,” Journal of Vascular and Interventional Radiology 24 (2013): 1808–1816. 24099787 10.1016/j.jvir.2013.08.018

[16] J. W. Chung , “Safety, Efficacy, and Response Predictors of Anticoagulation for the Treatment of Nonmalignant Portal‐Vein Thrombosis in Patients With Cirrhosis: A Propensity Score Matching Analysis,” Clinical and Molecular Hepatology 20 (2014): 384–391. 25548745 10.3350/cmh.2014.20.4.384 PMC4278070

[17] H. Chen , L. Liu , X. Qi , et al., “Efficacy and Safety of Anticoagulation in More Advanced Portal Vein Thrombosis in Patients With Liver Cirrhosis,” European Journal of Gastroenterology & Hepatology 28 (2016): 82–89. 26513611 10.1097/MEG.0000000000000482

[18] B. Scheiner , P. R. Stammet , S. Pokorny , et al., “Anticoagulation in Non‐Malignant Portal Vein Thrombosis Is Safe and Improves Hepatic Function,” Wiener Klinische Wochenschrift 130 (2018): 446–455. 29916054 10.1007/s00508-018-1351-y PMC6061656

[19] H. Hidaka , S. Kokubu , T. Sato , et al., “Antithrombin III for Portal Vein Thrombosis in Patients With Liver Disease: A Randomized, Double‐Blind, Controlled Trial,” Hepatology Research 48 (2018): E107–E116. 28666312 10.1111/hepr.12934

[20] A. A. Sule , J. B. Joseph , S. C. J. Chew , J. George , and T. J. Chin , “Exploring the Outcomes of Portal Vein Thrombosis in the Clinical Setting of Cirrhosis, Malignancy, and Intra‐Abdominal Infections With and Without Anticoagulation: A Retrospective 5‐Year Study,” International Journal of Angiology 27 (2018): 208–212. 10.1055/s-0037-1607049 PMC6221800 30410292

[21] C. Noronha Ferreira , D. Reis , H. Cortez‐Pinto , et al., “Anticoagulation in Cirrhosis and Portal Vein Thrombosis Is Safe and Improves Prognosis in Advanced Cirrhosis,” Digestive Diseases and Sciences 64 (2019): 2671–2683. 30852769 10.1007/s10620-019-05572-z

[22] T. Malek Mahmoudi , V. Marquez , A. Kayal , R. Carvalho , and A. A. Weiss , “HCC Complicated by PVT: Outcome and the Role of Anticoagulation Therapy,” Canadian Liver Journal 2 (2019): 121–126. 35990224 10.3138/canlivj.2018-0026 PMC9202748

[23] I. Pettinari , R. Vukotic , H. Stefanescu , et al., “Clinical Impact and Safety of Anticoagulants for Portal Vein Thrombosis in Cirrhosis,” American Journal of Gastroenterology 114 (2019): 258–266. 30538290 10.1038/s41395-018-0421-0

[24] M. H. Ai , W. G. Dong , X. P. Tan , et al., “Efficacy and Safety Study of Direct‐Acting Oral Anticoagulants for the Treatment of Chronic Portal Vein Thrombosis in Patients With Liver Cirrhosis,” European Journal of Gastroenterology & Hepatology 32 (2020): 1395–1400. 32675774 10.1097/MEG.0000000000001846

[25] T. Zhou , X. Sun , T. Zhou , et al., “Efficacy and Safety of Nadroparin Calcium‐Warfarin Sequential Anticoagulation in Portal Vein Thrombosis in Cirrhotic Patients: A Randomized Controlled Trial,” Clinical and Translational Gastroenterology 11, no. 9 (2020): e00228. 32858573 10.14309/ctg.0000000000000228 PMC7455225

[26] M. Florescu , A. Costache , S. Iacob , et al., “Anticoagulation Therapy for Portal Vein Thrombosis in Patients With Cirrhosis in a Tertiary Center Experience,” Journal of Gastrointestinal and Liver Diseases 30 (2021): 374–379. 34551038 10.15403/jgld-3392

[27] L. Naymagon , D. Tremblay , N. Zubizarreta , E. Moshier , J. Mascarenhas , and T. Schiano , “Safety, Efficacy, and Long‐Term Outcomes of Anticoagulation in Cirrhotic Portal Vein Thrombosis,” Digestive Diseases and Sciences 66 (2021): 3619–3629. 33151401 10.1007/s10620-020-06695-4

[28] Z. Gao , S. Li , J. Zhao , J. Li , and Y. Gao , “Anticoagulation Therapy Early Is Safe in Portal Vein Thrombosis Patients With Acute Variceal Bleeding: A Multi‐Centric Randomized Controlled Trial,” Internal and Emergency Medicine 18 (2023): 513–521. 36692588 10.1007/s11739-023-03206-x

[29] Z. I. Tarar , U. Farooq , F. Kamal , et al., “Safety of Anticoagulation Use for Treatment of Portal Vein Thrombosis in Liver Cirrhosis and Its Effect on Hospital‐Based Outcomes: An Insight From a US Nationwide Database,” Postgraduate Medical Journal 99 (2023): 715–723. 10.1136/pmj-2022-141915 37160356

[30] Z. Zhang , Y. Zhao , D. Li , et al., “Safety, Efficacy and Prognosis of Anticoagulant Therapy for Portal Vein Thrombosis in Cirrhosis: A Retrospective Cohort Study,” Thrombosis Journal 21 (2023): 1–8. 36717831 10.1186/s12959-023-00454-x PMC9885579

[31] A. Mantaka , N. Gatselis , C. K. Triantos , et al., “Treatment of Portal Vein Thrombosis in Cirrhosis: A Multicenter Real Life Cohort Study,” Minerva Gastroenterologica 69 (2023): 107–113. 10.23736/S2724-5985.21.02861-8 36856274

[32] A. Sato , S. Watanabe , M. Iseki , et al., “Anticoagulation Against Portal Vein Thrombosis Reduces Mortality and Liver Cirrhosis‐Related Complications: A Propensity Score‐Matched Study,” Hepatology Research 53 (2023): 1096–1104. 37435880 10.1111/hepr.13942

[33] S. Lu , J. Chen , R. Zhang , et al., “Comparative Effectiveness of Warfarin in Cirrhotic Patients With Non‐Symptomatic Portal Vein Thrombosis: A Multicenter, Randomized Controlled Trial,” Expert Review of Gastroenterology & Hepatology 18 (2024): 5–12. 38236640 10.1080/17474124.2024.2307575

[34] C. Niu , J. Zhang , K. Himal , et al., “Impact of Anticoagulation Therapy on Outcomes in Patients With Cirrhosis and Portal Vein Thrombosis: A Large‐Scale Retrospective Cohort Study,” Thrombosis Research 241 (2024): 109103. 39067278 10.1016/j.thromres.2024.109103

[35] C. Zhan , V. Prabhu , S. K. Kang , et al., “Comparison of Non‐Tumoral Portal Vein Thrombosis Management in Cirrhotic Patients: Tips Versus Anticoagulation Versus no Treatment,” Journal of Clinical Medicine 10 (2021): 2316. 34073236 10.3390/jcm10112316 PMC8198761

[36] A. Guerrero , L. del Campo , F. Piscaglia , et al., “Anticoagulation Improves Survival in Patients With Cirrhosis and Portal Vein Thrombosis: The IMPORTAL Competing‐Risk Meta‐Analysis,” Journal of Hepatology 79 (2023): 69–78. 36858157 10.1016/j.jhep.2023.02.023

[37] M. Biolato , M. Paratore , L. Di Gialleonardo , G. Marrone , and A. Grieco , “Direct Oral Anticoagulant Administration in Cirrhotic Patients With Portal Vein Thrombosis: What Is the Evidence?,” World Journal of Hepatology 14 (2022): 682–695. 35646264 10.4254/wjh.v14.i4.682 PMC9099104

[38] K. Martens , H. S. McMurry , S. Koprowski , et al., “Anticoagulation in Cirrhosis: Evidence for the Treatment of Portal Vein Thrombosis and Applications for Prophylactic Therapy,” Journal of Clinical Gastroenterology 56 (2022): 536–545. 35537133 10.1097/MCG.0000000000001713 PMC9189067

[39] Z. Zhang , Y. Zhao , B. Han , Z. Zhu , L. Sun , and X. Cui , “The Efficacy and Safety of Anticoagulants in the Treatment of Cirrhotic Portal Vein Thrombosis: A Systematic Review and Meta‐Analysis,” Clinical and Applied Thrombosis/Hemostasis 28 (2022). 10.1177/10760296221104797 PMC9168872 35656719

[40] C. Yao , M. Zhao , B. Ibrahim , and S. Saab , “Anticoagulation for the Treatment of Portal Vein Thrombosis in Cirrhosis: A Systematic Review and Meta‐Analysis of Comparative Studies,” Journal of Clinical and Experimental Hepatology 13 (2023): 404–413. 37250883 10.1016/j.jceh.2022.12.016 PMC10213860

[41] J. G. O'Leary , C. S. Greenberg , H. M. Patton , and S. H. Caldwell , “AGA Clinical Practice Update: Coagulation in Cirrhosis,” Gastroenterology 157 (2019): 34–43e1. 30986390 10.1053/j.gastro.2019.03.070

[42] Y. Shi , W. Feng , J. Cai , et al., “Analysis of Factors Related to Recanalization of Portal Vein Thrombosis in Liver Cirrhosis: A Retrospective Cohort Study,” BMC Gastroenterology 24 (2024): 1–12. 39003447 10.1186/s12876-024-03322-8 PMC11245851

[43] J. C. Garcia‐Pagán , E. Buscarini , H. L. A. Janssen , et al., “EASL Clinical Practice Guidelines: Vascular Diseases of the Liver,” Journal of Hepatology 64 (2016): 179–202. 26516032 10.1016/j.jhep.2015.07.040

[44] A. Boccatonda , S. Gentilini , E. Zanata , et al., “Portal Vein Thrombosis: State‐Of‐The‐Art Review,” Journal of Clinical Medicine 13 (2024): 1517. 38592411 10.3390/jcm13051517 PMC10932352

[45] X. Qi , V. De Stefano , H. Li , J. Dai , X. Guo , and D. Fan , “Anticoagulation for the Treatment of Portal Vein Thrombosis in Liver Cirrhosis: A Systematic Review and Meta‐Analysis of Observational Studies,” European Journal of Internal Medicine 26 (2015): 23–29. 25566699 10.1016/j.ejim.2014.12.002

[46] A. S. Wolberg and Y. Sang , “Fibrinogen and Factor XIII in Venous Thrombosis and Thrombus Stability,” Arteriosclerosis, Thrombosis, and Vascular Biology 42 (2022): 931–941. 35652333 10.1161/ATVBAHA.122.317164 PMC9339521

[47] A. Zaman, MM , “Anticoagulation for Portal Vein Thrombosis in Patients With Cirrhosis,” NEJM Journal Watch (2019).

[48] H. Samant , K. O. Asafo‐Agyei , A. Kimyaghalam , and K. Garfield , “Portal Vein Thrombosis,” StatPearls (2024). 30480933

[49] L. Loffredo , D. Pastori , A. Farcomeni , and F. Violi , “Effects of Anticoagulants in Patients With Cirrhosis and Portal Vein Thrombosis: A Systematic Review and Meta‐Analysis,” Gastroenterology 153 (2017): 480–487.e1. 28479379 10.1053/j.gastro.2017.04.042

[50] Y. Gao , H. Liu , F. Tang , et al., “Efficacy and Safety of Anticoagulants in Liver Cirrhosis Patients With Portal Vein Thrombosis: A Meta‐Analysis,” Clinical Research in Hepatology and Gastroenterology 45, no. 2 (2021): 101649. 10.1016/j.clinre.2021.101649 33601064

[51] A. Cuker , A. Burnett , D. Triller , et al., “Reversal of Direct Oral Anticoagulants: Guidance From the Anticoagulation Forum,” American Journal of Hematology 94 (2019): 697–709. 30916798 10.1002/ajh.25475

[52] D. A. Simonetto , A. K. Singal , G. Garcia‐Tsao , S. H. Caldwell , J. Ahn , and P. S. Kamath , “ACG Clinical Guideline: Disorders of the Hepatic and Mesenteric Circulation,” American Journal of Gastroenterology 115 (2020): 18–40. 31895720 10.14309/ajg.0000000000000486

[53] C. T. Ruff , R. P. Giugliano , E. Braunwald , et al., “Comparison of the Efficacy and Safety of New Oral Anticoagulants With Warfarin in Patients With Atrial Fibrillation: A Meta‐Analysis of Randomised Trials,” Lancet 383 (2014): 955–962. 24315724 10.1016/S0140-6736(13)62343-0

