# Supplementary material for: Efficacy and Safety of Anticoagulants in Patients With Cirrhosis and Portal Vein Thrombosis: A Systematic Review and Meta‐Analysis of Randomized and Non‐Randomized Studies
Source: JGH Open. 2025 Aug 8;9(8):e70194. doi: 10.1002/jgh3.70194 (PMC12333073; doi:10.1002/jgh3.70194)
Supplement: Supplementary file 1 — Table S1. Detailed Search Strategies Used in Different Databases. Table S2. Newcastle Ottawa Scale for the quality assessment of the non‐randomized studies. Figure S1. Quality assessment of the RCTs by RoB 2.0 tool. Figure S2. Subgroup analysis based on anticoagulant type for PVT recanalization. Figure S3. Subgroup analysis based on anticoagulant type for PVT improvement. Figure S4. Subgroup analysis based on anticoagulant type for PVT stability. Figure S5. Subgroup analysis based on anticoagulant type for PVT progression. Figure S6. Subgroup analysis based on anticoagulant type for mortality. Figure S7. Total Bleeding Forest plot. Figure S8. Subgroup analysis based on anticoagulant type for total bleeding. Figure S9. Esophageal variceal bleeding Forest plot. Figure S10. Subgroup analysis based on anticoagulant type for Esophageal variceal bleeding. Figure S11. Gastrointestinal Bleeding Forest plot. Figure S12. Subgroup analysis based on anticoagulant type for GI bleeding. Figure S13. Intracranial Hemorrhage Forest plot. Figure S14. Subgroup analysis based on anticoagulant type for intracranial hemorrhage. Figure S15. Esophageal variceal bleeding leave‐one‐out sensitivity analysis plot. Figure S16. Mortality leave‐one‐out sensitivity analysis plot. Figure S17. Portal Vein Thrombosis Recanalization Funnel plot. Figure S18. Portal Vein Thrombosis Improvement Funnel plot. Figure S19. Portal Vein Thrombosis Stability Funnel plot. Figure S20. Portal vein thrombosis progression Funnel plot. Figure S21. Mortality Funnel plot. Figure S22. Total Bleeding Funnel plot. Figure S23. Esophageal variceal bleeding Funnel plot. Figure S24. Gastrointestinal Bleeding Funnel plot. Figure S25. Intracranial Hemorrhage Funnel plot. [file JGH3-9-e70194-s001.docx]

| **Supplementary Table S1:** Detailed Search Strategies Used in Different Databases | | |
| --- | --- | --- |
| **Database** | **Search String** | **Articles Retrieved** |
| PubMed | ("Liver Cirrhosis"[Mesh] OR Cirrhosis, Liver OR Hepatic Cirrhosis OR Cirrhosis, Hepatic OR Fibrosis, Liver OR Liver Fibrosis) AND ("Anticoagulants"[Mesh] OR Anticoagulant Drug OR Drug, Anticoagulant OR Anticoagulant Agent OR Agent, Anticoagulant OR Anticoagulation Agents OR Agents, Anticoagulation OR Anticoagulant Drugs OR Drugs, Anticoagulant OR Anticoagulant Agents OR Agents, Anticoagulant OR Anticoagulant OR Indirect Thrombin Inhibitors OR Inhibitors, Indirect Thrombin OR Thrombin Inhibitors, Indirect) AND (Portal vein thrombosis OR Portal venous thrombosis OR PVT) | 587 |
| Cochrane Central | ("Liver Cirrhosis"[Mesh] OR Cirrhosis, Liver OR Hepatic Cirrhosis OR Cirrhosis, Liver Fibrosis) AND ("Anticoagulants"[Mesh] OR Anticoagulant Drug OR Drug, Anticoagulant OR Anticoagulation Agents OR Agents, Anticoagulation OR Indirect Thrombin Inhibitors OR Thrombin Inhibitors, Indirect) AND (Portal vein thrombosis OR Portal venous thrombosis OR PVT) | 55 |
| ScienceDirect | ("Liver Cirrhosis" OR "Hepatic Cirrhosis" OR "Liver Fibrosis") AND ("Anticoagulants" OR "Anticoagulant Drugs" OR "Indirect Thrombin Inhibitors") AND ("Portal Vein Thrombosis" OR "Portal Venous Thrombosis" OR "PVT") | 821 |

| **Supplementary Table S2:** Newcastle Ottawa Scale for the quality assessment of the non-randomized studies | | | | | | | | | | |
| --- | --- | --- | --- | --- | --- | --- | --- | --- | --- | --- |
| **Cohort Studies** | | **Selection** | | | | **Comparability** | **Exposure** | | | **Total** |
| **Author** | **Year** | **Representativeness of Exposed Cohort** | **Selection of the Non exposed cohort** | **Ascertainment of Exposure** | **Outcome was not Present at the start of Study** |  | **Assessment of Outcome** | **Follow-up long enough for Outcomes to occur** | **Adequacy of follow-up of Cohorts** |  |
| Ai 2020 | 2020 | ******* | ******* | ******* | ******* | ******* | ******* | ******* | ******* | 8 |
| Florescu 2021 | 2021 | ******* | ******* | ******* | ******* | ******* | ******* | ******* | ******* | 8 |
| Naymagon 2021 | 2021 | ******* | ******* | ******* | ******* | ******* | ******* | ******* | ******* | 8 |
| Zhan 2021 | 2021 | ******* | ******* | ******* | ******* | ******** | ******* | ******* | ******* | 9 |
| Tarar 2022 | 2022 | ******* | ******* | ******* | ******* | ******* | ******* |  |  | 6 |
| Zhang 2023 | 2023 | ******* | ******* | ******* | ******* | ******* | ******* | ******* | ******* | 8 |
| MANTAKA 2023 | 2023 | ******* | ******* | ******* | ******* | ******* | ******* | ******* | ******* | 8 |
| Sato 2023 | 2023 | ******* | ******* | ******* | ******* | ******* | ******* |  |  | 6 |
| Niu 2024 | 2024 | ******* | ******* | ******* | ******* | ******* | ******* |  |  | 6 |
| Senzolo 2012 | 2012 | ******* | ******* | ******* |  | ******* | ******* |  | ******* | 6 |
| Chung 2014 | 2014 | ******* | ******* | ******* | ******* | ******* | ******* | ******* |  | 7 |
| Chen 2016 | 2016 | ******* | ******* | ******* | ******* | ******* | ******* | ******* |  | 7 |
| Scheiner 2018 | 2018 | ******* | ******* | ******* |  | ******* | ******* | ******* | ******* | 7 |
| Sule 2018 | 2018 | ******* | ******* | ******* | ******* | ******* | ******* |  |  | 6 |
| Acuna-Villaorduna 2019 | 2019 | ******* | ******* | ******* | ******* | ******* | ******* |  | ******* | 7 |
| Mahmoudi 2019 | 2019 | ******* | ******* | ******* | ******* | ******* | ******* |  |  | 6 |
| Pettinari 2019 | 2019 | ******* | ******* | ******* | ******* | ******* | ******* |  |  | 6 |
| Cai 2013 | 2013 | ******* | ******* | ******* | ******* | ******* | ******* | ******* | ******* | 8 |
| Ferreira 2019 | 2019 | ******* | ******* | ******* | ******* | ******* | ******* |  |  | 6 |


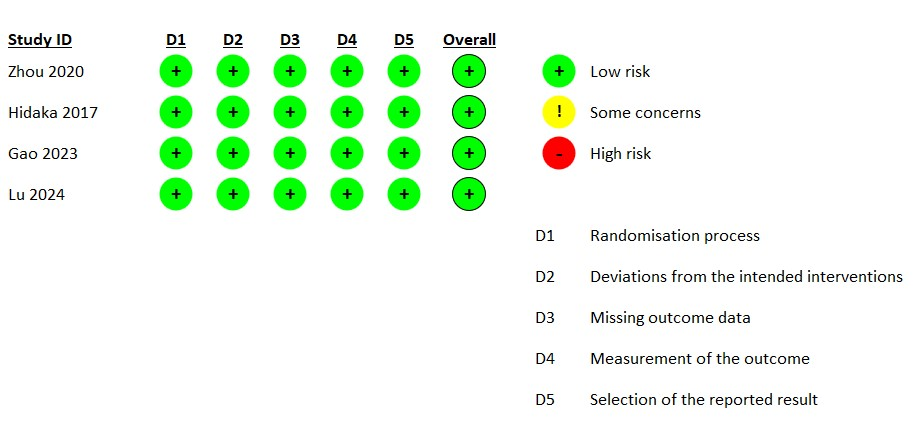


**Supplementary Figure S1:** Quality assessment of the RCTs by RoB 2.0 tool


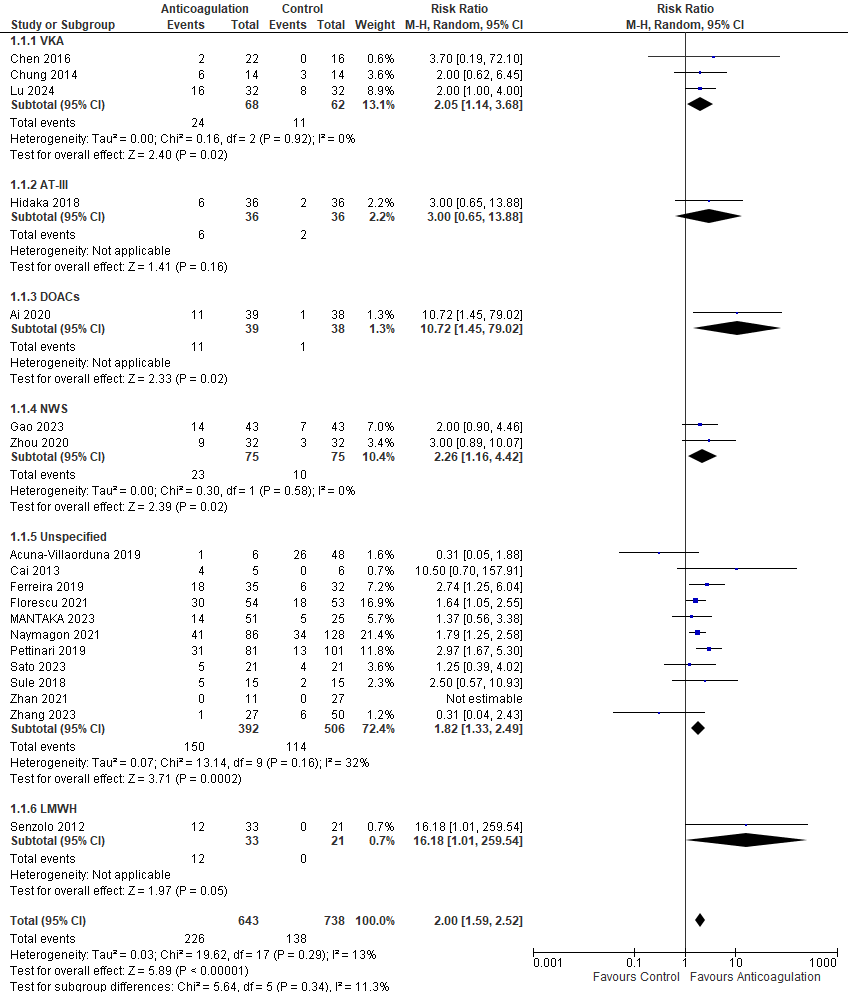


**Supplementary Figure S2:** Subgroup analysis based on anticoagulant type for PVT recanalization


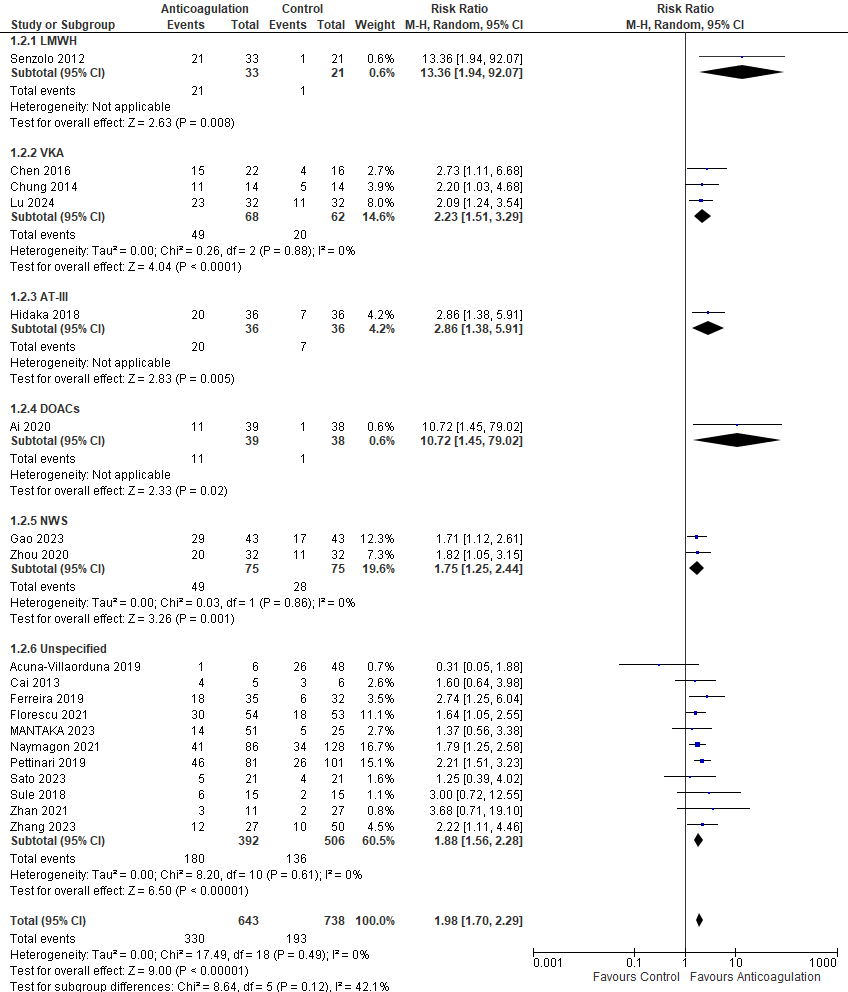


**Supplementary Figure S3:** Subgroup analysis based on anticoagulant type for PVT improvement


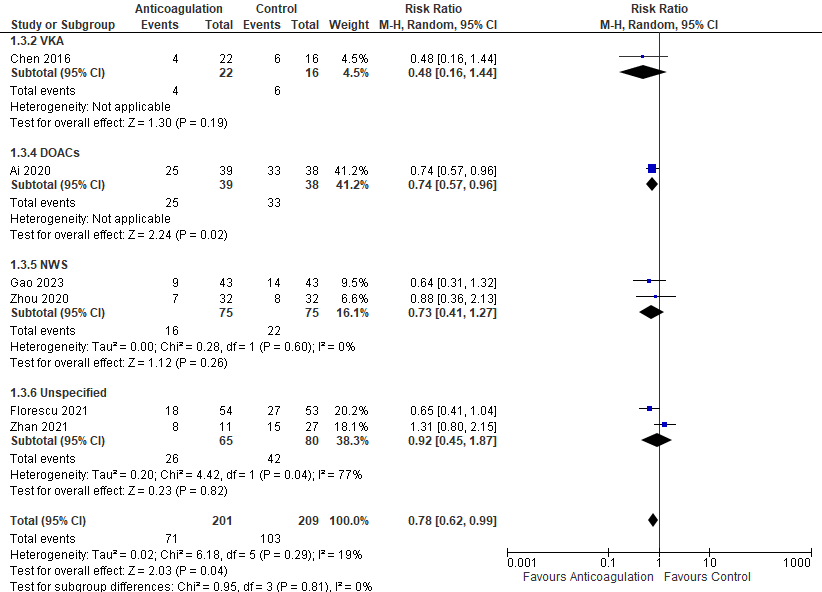


**Supplementary Figure S4:** Subgroup analysis based on anticoagulant type for PVT stability

**
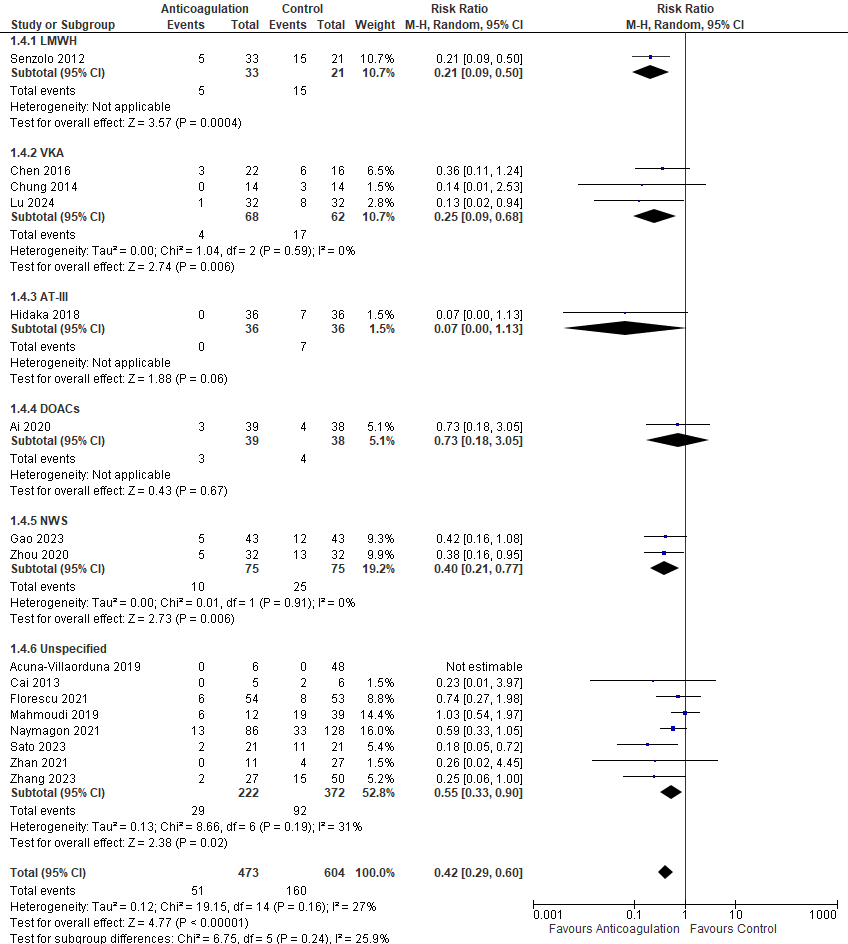
**

**Supplementary Figure S5:** Subgroup analysis based on anticoagulant type for PVT progression


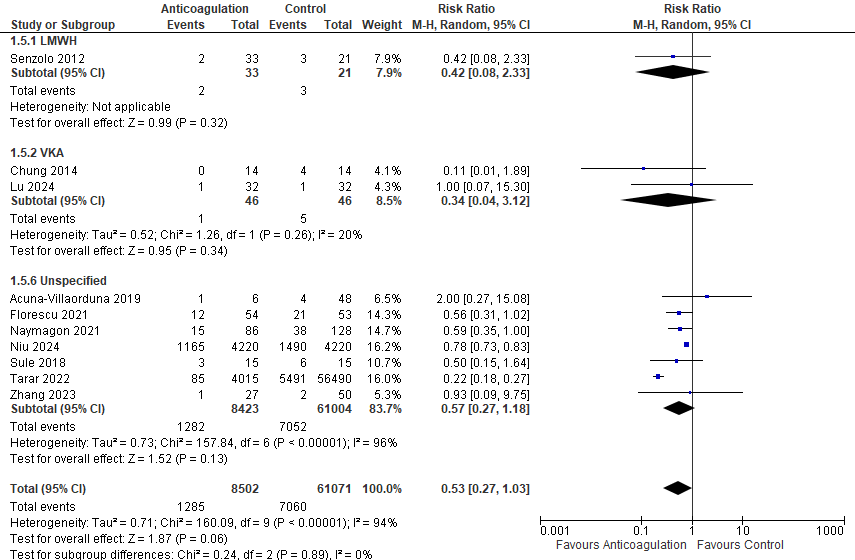


**Supplementary Figure S6:** Subgroup analysis based on anticoagulant type for mortality


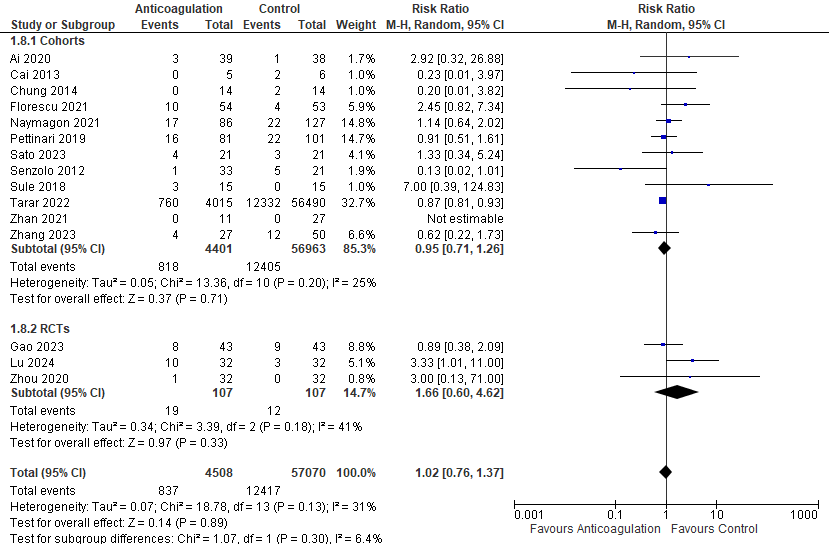


**Supplementary Figure S7:** Total Bleeding Forest plot

**
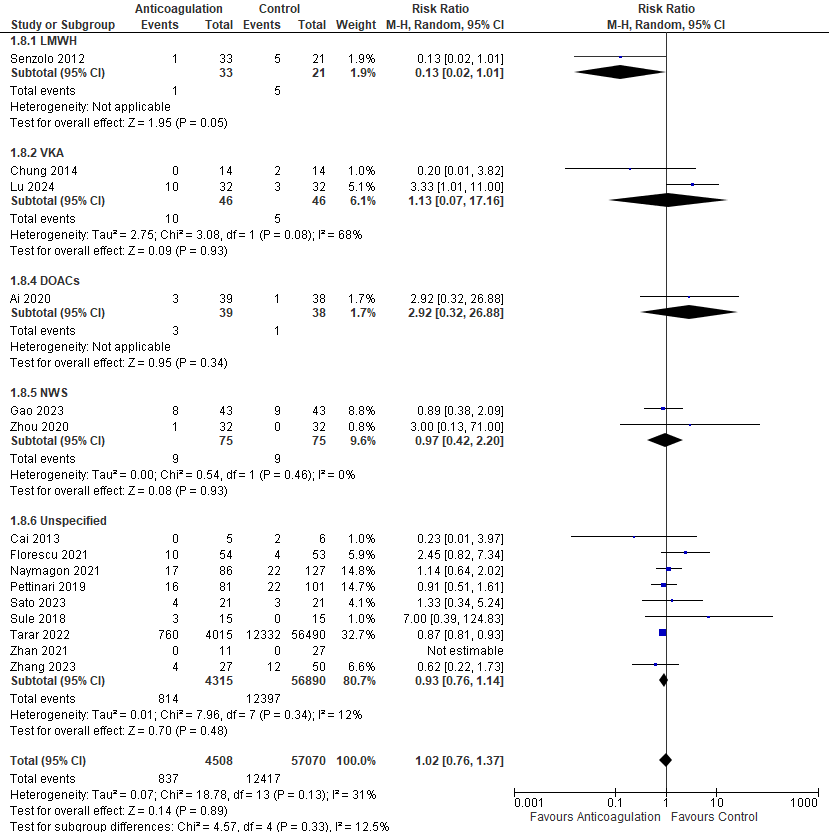
**

**Supplementary Figure S8:** Subgroup analysis based on anticoagulant type for total bleeding


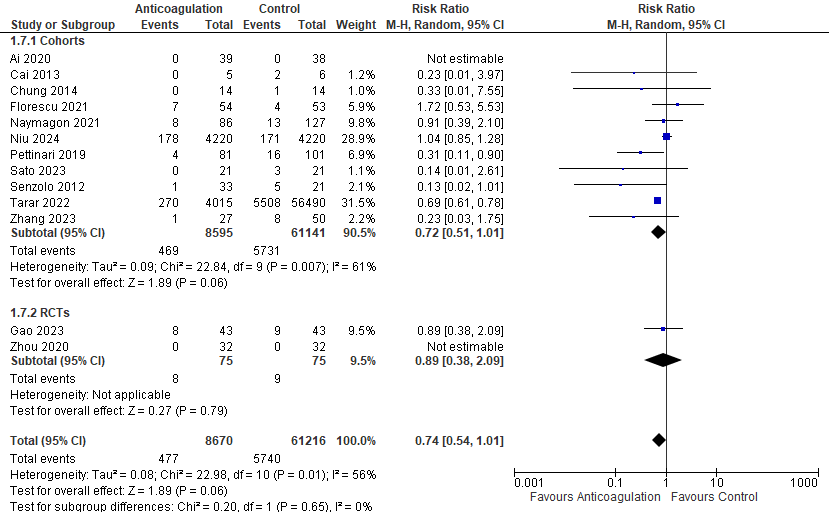


**Supplementary Figure S9**: Esophageal variceal bleeding Forest plot

**
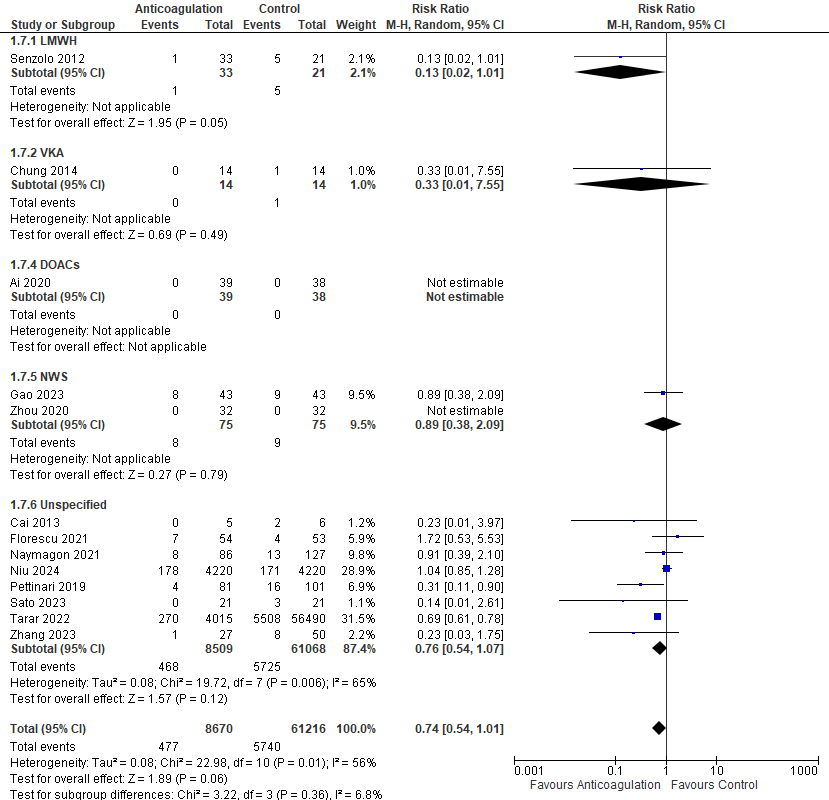
**

**Supplementary Figure S10:** Subgroup analysis based on anticoagulant type for Esophageal variceal bleeding


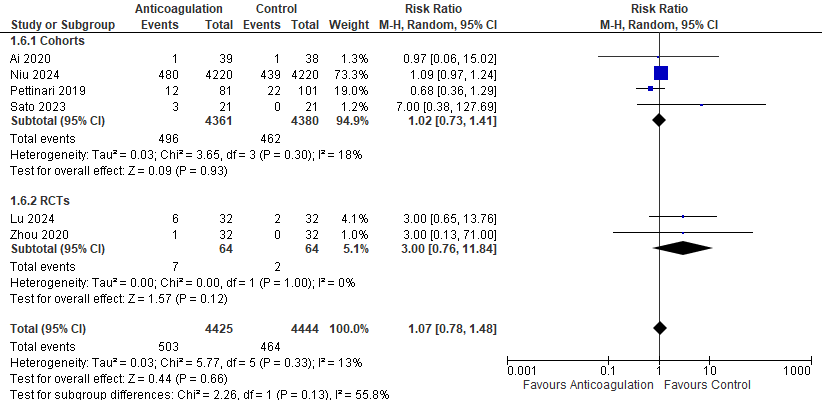


**Supplementary Figure S11:** Gastrointestinal Bleeding Forest plot


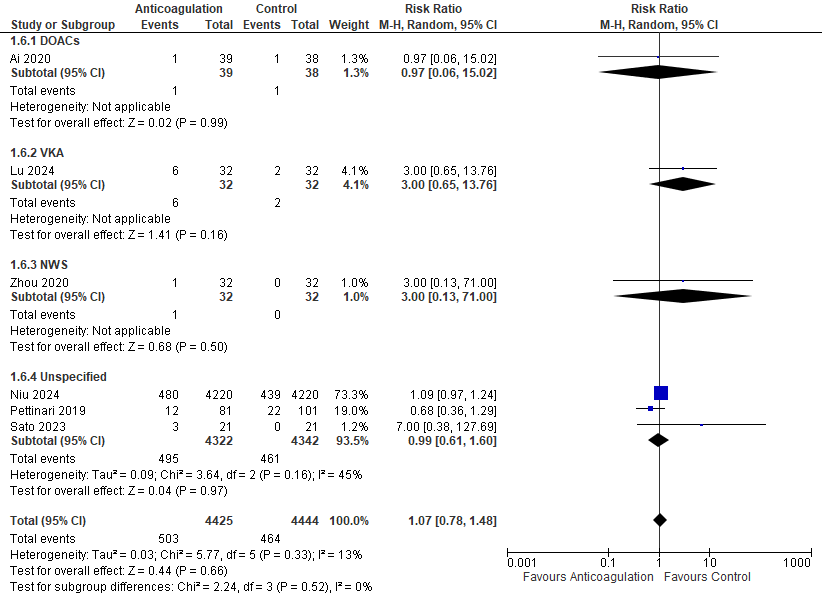


**Supplementary Figure S12:** Subgroup analysis based on anticoagulant type for GI bleeding


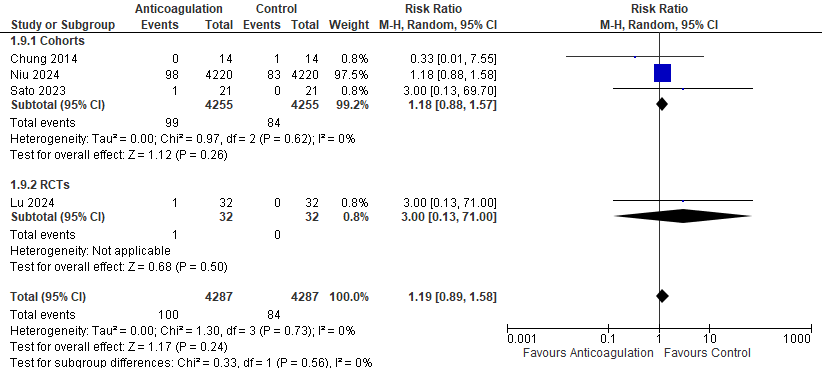


**Supplementary Figure S13:** Intracranial Hemorrhage Forest plot


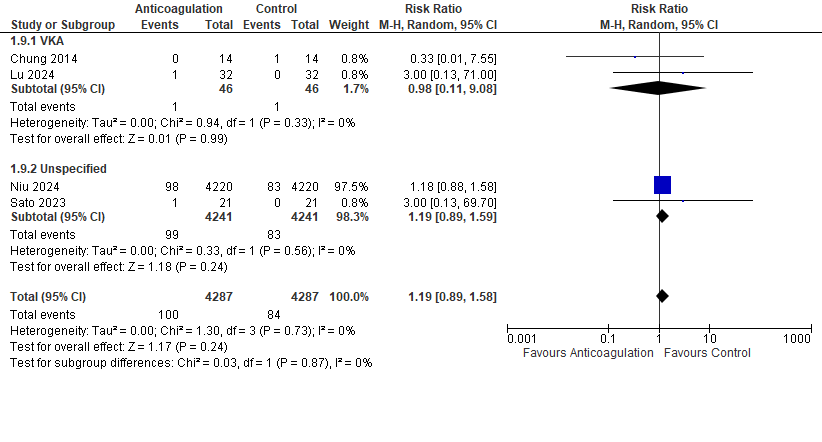


**Supplementary Figure S14:** Subgroup analysis based on anticoagulant type for intracranial hemorrhage


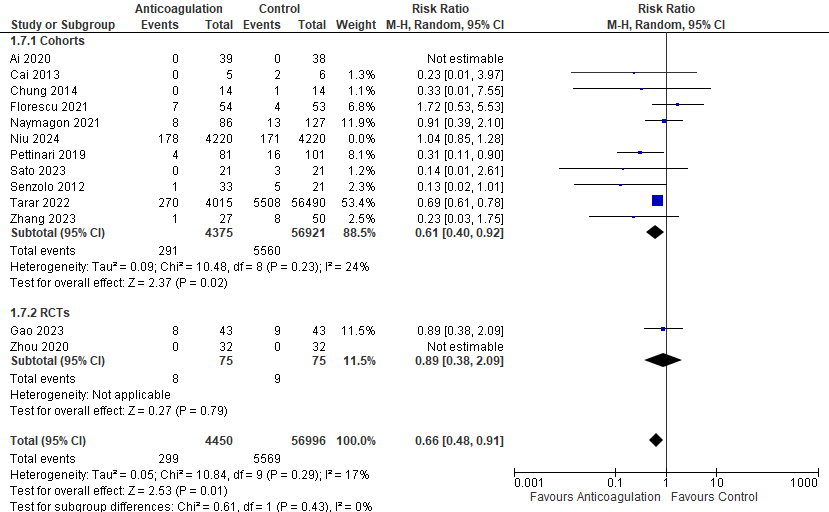


**Supplementary Figure S15:** Esophageal variceal bleeding leave-one-out sensitivity analysis plot


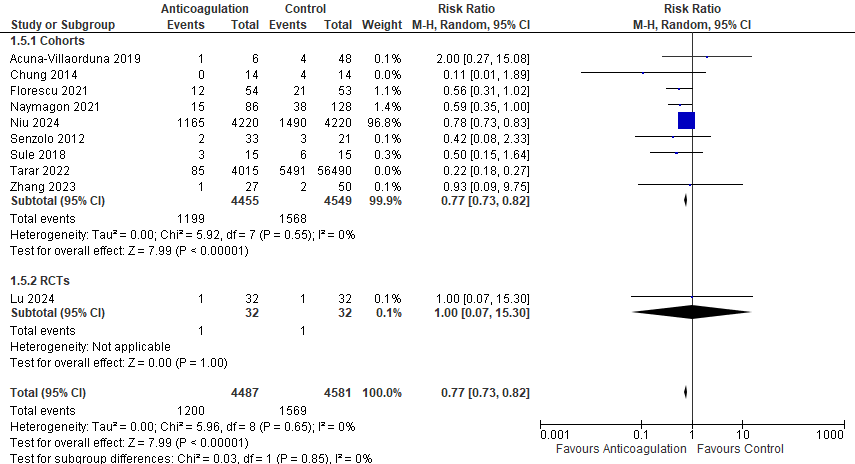


**Supplementary Figure S16:** Mortality leave-one-out sensitivity analysis plot


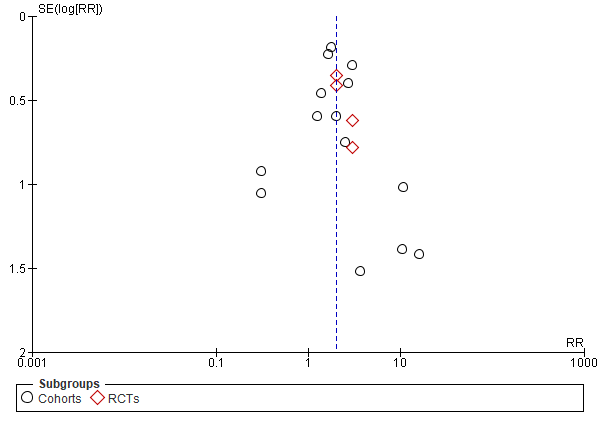


**Supplementary Figure S17:** Portal Vein Thrombosis Recanalization Funnel plot


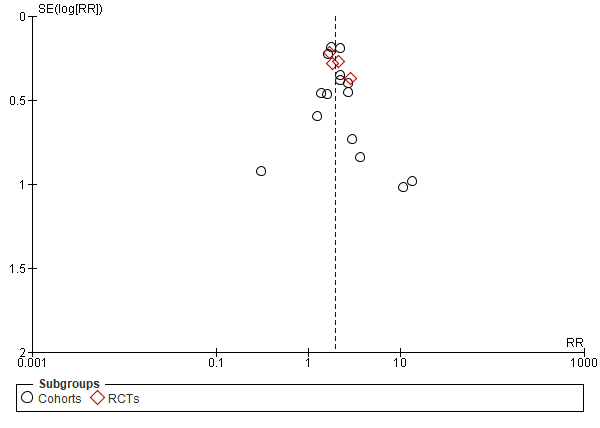


**Supplementary Figure S18:** Portal Vein Thrombosis Improvement Funnel plot


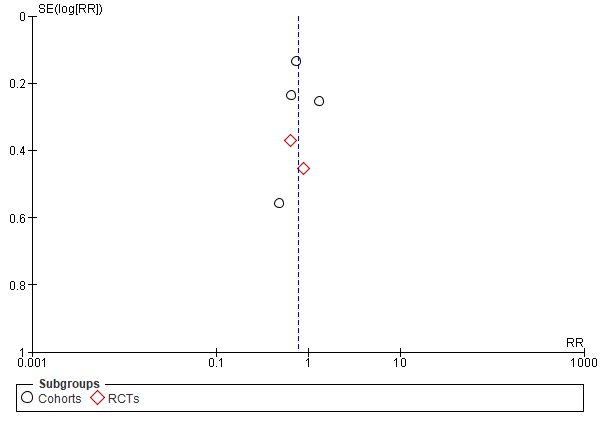


**Supplementary Figure S19:** Portal Vein Thrombosis Stability Funnel plot


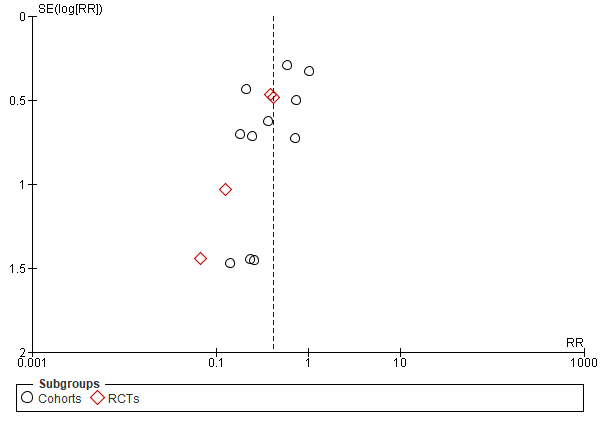


**Supplementary Figure S20**: Portal vein thrombosis progression Funnel plot


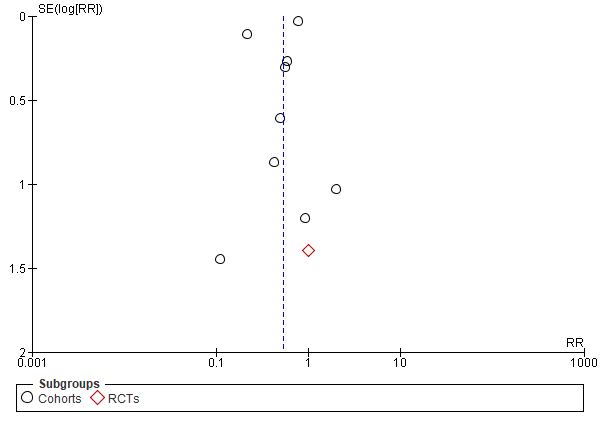


**Supplementary Figure S21:** Mortality Funnel plot

**
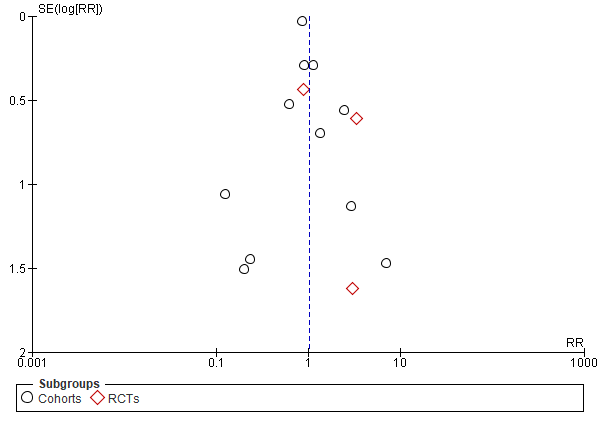
**

**Supplementary Figure S22**: Total Bleeding Funnel plot


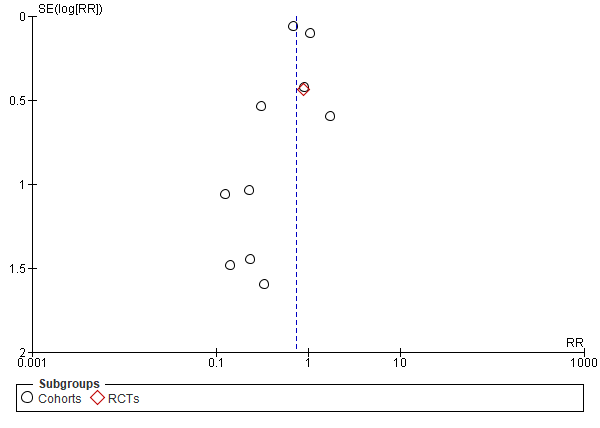


**Supplementary Figure S23:** Esophageal variceal bleeding Funnel plot


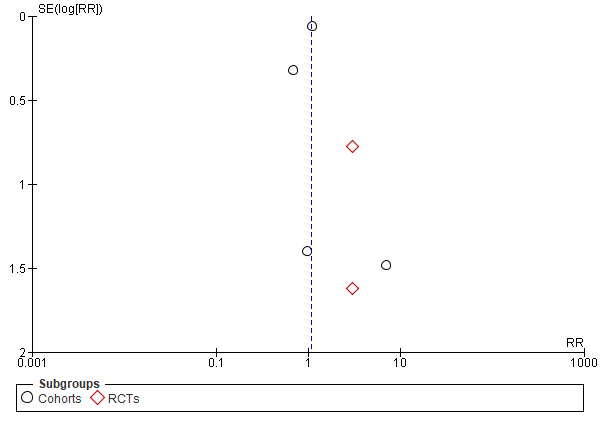


**Supplementary Figure S24:** Gastrointestinal Bleeding Funnel plot


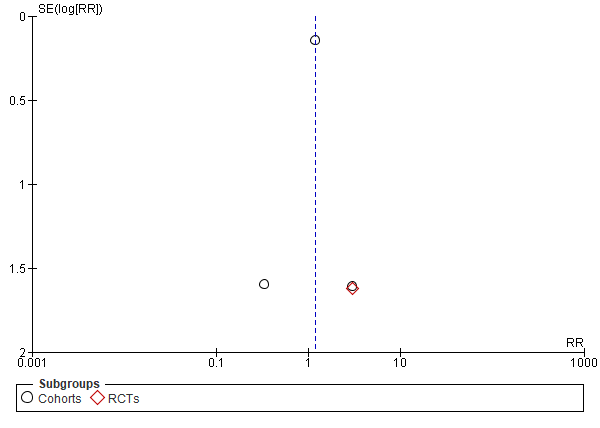


**Supplementary Figure S25:** Intracranial Hemorrhage Funnel plot
